# Identifying Adaptable Varieties of Sorghum ( *Sorghum bicolor *L) in Tidal Swamplands and Sandy Soils by MGIDI and GGE Biplots

**DOI:** 10.12688/f1000research.166848.2

**Published:** 2025-11-06

**Authors:** Susilawati Susilawati, Muhamad Sabran, Twenty Liana, Suwardi Suwardi, Retna Qomariah, Susi Lesmayati, Andy Bhermana, Dwi P Widiastuti, YantiRina Darsani

**Affiliations:** 1 Research Center for Food Crops, Agriculture and Food Research Organization, National Research and Innovation Agency, Cibinong, 16911, Indonesia; 2 Research Center for Behavioral and Circular Economics, Governance,Economic, and Community Welfare Research Organization-National Research and Innovation Agency, Jakarta, 12710, Indonesia; 3 Research Center for Agroindustry, Agriculture and Food Research Organization-National Research and Innovation Agency, Tangerang Selatan, 15310, Indonesia

**Keywords:** Varieties, sorghum, adaptable, tidal swamplands, sandy soil, MGIDI, GGE biplot.

## Abstract

**Background:**

Sorghum has potential as a source of material for food, bioenergy, and animal feed, making it a worthy candidate for promotion. This cereal thrives in regions characterized by low moisture and dry conditions. To address the diminishing availability of arable dry land, it may be necessary to explore the cultivation of sorghum in tidal swamplands and sandy soils.

**Methods:**

Twelve sorghum varieties were evaluated in tidal swamplands during the rainy and dry seasons, as well as in sandy soil during the dry season, using two levels of organic fertilizers to create six test environments. The experiments were arranged in a completely randomized block design with three replications. To choose sorghum varieties with features that closely resemble an idealized sorghum variety, the Multi-trait Genotype-Ideotype Distance Index (MGIDI) was utilized. Simultaneously, genotype plus genotype-environment interaction (GGE) biplots were employed to determine the best circumstances for choosing broadly adaptable varieties that exhibit desirable features, as well as to find varieties that thrive environmental contexts.

**Results:**

Based on the MGIDI ranking on the average across environment, two varieties, i.e., *Numbu* and *Kawali* were selected. However selected varieties in each environment were differed due to significant variety-environment interaction. In terms of grain weight, the *Soper 7 Agritan* variety exhibits adaptability across diverse environments, while the *Numbu* variety likewise demonstrates versatility in various environmental conditions. When evaluating forage yield, several adaptable varieties have emerged. Tidal swamplands treated with a high application of organic fertilizer, as well as sandy soils, provide optimal environments for selecting broadly adaptable varieties that focus on both grain and forage yields.

**Conclusion:**

Adaptable varieties differ for various groups of environments and different traits under consideration. Optimal environments for identifying broadly adaptable varieties varied by trait. The MGIDI proves to be a valuable tool for selecting varieties based on multiple traits. In parallel, the GGE biplots effectively identifies adaptable varieties based on individual traits.

## 1. Introduction

The sorghum crop ( *Sorghum bicolor* L.) plays a significant role as a source of food, bioenergy, and animal feed materials. As a food source, it provides carbohydrate sources and other essential nutrients, including proteins, polyunsaturated fatty acids, and high fiber. The utilization of sorghum can then be promoted for food diversification. ^1, 2^ As a source of bioenergy, it produces biomass that can be processed through fermentation, gasification, and fast pyrolysis to generate various biofuels, including bioethanol, biodiesel, bio-oil, biogas, biohydrogen, and other bio-derived products. ^3– 6^ Sorghum also serves as a source of feed for animals.

Sorghum ( *Sorghum bicolor* L.) is a highly adaptable crop that thrives in diverse agroecosystems due to its genetic diversity and resilience to various environmental stresses. ^7– 9^ Sorghum crops are primarily cultivated in drylands due to their drought resistance, which is attributed to their evolution in arid regions. As a drought-resistant crop, sorghum is widely cultivated in many areas, including semi-arid and arid zones in Africa, Asia, the Middle East, Central America, North America, and Australia. ^4, 5, 10^

In Indonesia, sorghum is mainly cultivated in dry lands. However, the availability of dry lands for sorghum cultivation continually reduced due to land conversion for non-agricultural purposes and competition with other crops, prompting the need to expand sorghum cultivation to tidal swamplands and sandy soil areas, which are quite promising and widely available in Indonesia. It was estimated that 8.92 million hectares of tidal swamplands and 2.10 million hectares of sandy soils were available for agriculture in Indonesia. ^11, 12^

Swamplands are low-lying lands that are regularly flooded. It consists of two types of lands, i.e., tidal and inland swamplands. Tidal swamplands are swamplands that are influenced by sea tides. It can be further classified based on tidal influence into types A, B, C, and D. ^11^ Tidal swamplands of type A are those lands influenced by spring and neap tides, whereas type B are those influenced by neap tides only. Suppose there is no flooding, i.e., only a rise in the water table during the tides, then those lands are classified as type C, while type D is not influenced by sea tides at all, and thus, basically a dry land in the swampy areas. Inland swamps are areas formed in the inland valley where water originates from an upstream river or rainfall. Sandy soil contains a high proportion of sand particles, i.e., more than 60% of sand by volume, derived from sedimentary rock. It has a gritty texture, excellent drainage, poor nutrient retention, and good airflow. ^13^

The expansion of sorghum cultivation to tidal swamplands and sandy soils necessitates the development of varieties that can thrive in these environments. A crop’s adaptability is defined by its ability to grow and yield well under varying environmental conditions. Consequently, high phenotypic performance and consistency across different environments serve as critical indicators of adaptability. While sorghum’s adaptability has been investigated in dryland environments ^14– 18^—encompassing a range of climates from semi-arid and dry to humid, ^19, 20^ as well as ^21^ various agroclimatic conditions, ^22– 26^ differing altitudes, ^27^ and diverse fertilizer applications ^17, 28^—there is a notable lack of research focusing on sorghum’s response to high rainfall and inundation, as well as to nutrient-poor and pyrite-containing soils characteristic of tidal swamplands and sandy soils. This gap presents a valuable opportunity for further exploration. A recent 2023 study indicated that tidal swamplands could support sorghum cultivation, with soil acidity and limited nutrient availability ^29, 30^ identified as the primary challenges. Additional research is needed not only in swampy areas but also in other agroecosystems featuring sandy soils to assess the suitability of this crop for these environments.

Environmental and variety-based adaptation research, including the use of biofertilizers and nutrients, as well as the influence of climate on swampy and sandy soils, are phenomena that require study. Given that superior sorghum varieties can adapt or tolerate climate change or stress. Intercropping and integrated nutrient management, as well as land and water management practices, are key adaptations that can enhance the health and productivity of marginal soils and are effective in increasing sorghum yields. ^29^

The purposes of this research are: 1. to identify a high-performance variety based on multipl traits and beneficial characteristics in tidal swamplands and sandy soils. 2. To determine adaptable varieties in tidal swampland and sandy soil, and 3. To determine the best environment to test broadly adaptable varieties. The high-performance and adaptable varieties were selected using the Multi-Trait Genotype-Ideotype Distance Index (MGIDI) and Genotype plus Genotype vs Environment (GGE) biplot.

MGIDI is a tool for selecting plant genotypes and ranking agronomic treatments based on multiple traits. It integrates various traits into a single index. It could be used to select varieties and their interaction with an environment close to the ideal type of sorghum in tidal swamplands and sandy soils. ^31– 33^ MGIDI embedding weight to prioritize traits, reduce dimensionality, and enhance selection accuracy. ^32, 33^ Some studies have shown that MGIDI can lead to significant selection gains across various traits. ^34^ The GGE biplot is a graphical tool for studying the performance of varieties in multiple tested environments. The biplot illustrates the two factors (G and GE) that are important in variety evaluation. The GGE biplot displays the first two principal components (PC1 and PC2) derived from environment-centered data, i.e., when the effect of environment is removed from the multi-environment data of the cultivar. This method has been employed in numerous studies to investigate adaptability and genotype-environment interaction in sorghum. ^35– 50^

## 2. Materials and methods

### 2.1 Experimental sites

The experiments were conducted from October 2022 to February 2023 (wet season) and from July to November 2024 (dry season) in tidal swamplands at *Petak Batuah* Village, *Dadahup* Sub-district, *Kapuas* Regency, and from August to December 2023 (dry season) in sandy soils at *Sidodadi* Village, *Bukit Batu* Sub-district, *Palangka Raya* City, Central Kalimantan Province, Indonesia.

### 2.2 Plant material

This study used 12 varieties of sorghum (  Table 1). The Cereal Crop Instrument Standard Testing Centre (CCISTC) is the source of all seeds.  Table 1 shows some of the main characteristics of these varieties. Other materials needed include soil conditioners such as dolomite and chicken manure. The inorganic fertilizers are Urea, NPK, SP-36, and KCl. Several insecticides were applied as required.

**Table 1.** The main characteristics of the tested sorghum varieties.

Varieties (code)	Origin	Pest and disease resistance ^*^	Plant age at 50% flowering (dap) ^**^	Carbohydrates (%) ^***^	Tanin (%)	Yield (t ha ^−1^)
Super 1 (V1)	CCISTC germplasm collection	*Aphis* (R), *Anthracnose*, leaf rust, and leaf blight (R)	56	71.30	0.110	5.70
Super 2 (V2)	Introduction from ICRISAT	*Aphis* (R), *Anthracnose*, leaf rust and leaf blight (R)	60	75.60	0.300	6.30
Suri 3 Agritan (V3)	Introduction from ICRISAT	*Aphis* (R), *Anthracnose* and leaf spot (R)	54	64.06	0.077	6.00
Suri 4 Agritan (V4)	Introduction from ICRISAT	*Aphis*, *Anthracnose*, and leaf spot (MR)	55	64.93	0.013	5.70
Mandau (V5)	Introduction from IRRI	stem borers (R), *Anthracnose* and leaf rust (R)	65	76.00	na	4.00–5.00
*Soper* 6 Agritan (V6)	Introduction from ICRISAT	*Aphis* and leaf rust (HR), leaf spot *, * and *Anthracnose* (MR)	64	66.88	0.070	6.00
*Soper* 7 Agritan (V7)	Crossing of *Numbu*/15011-B	leaf rust and leaf spot (R), *Anthracnose* and stem rot (HR)	59–65	63.90	0.210	12.93
*Numbu* (V8)	India	*Aphis* (R), leaf rust and leaf spot (R)	69	84.58	na	4.00–5.00
*Soper* 9 Agritan (V9)	Crossing 4-183-A/ *Numbu*	leaf rust (R), leaf spot, *Anthracnose*, and stem rot (HR)	62–65	63.86	0.210	14.40
Kawali (V10)	India	*Aphis* (MR), leaf rust and leaf spot (R)	70	87.87	na	4.00–5.00
Bioguma II Agritan (V11)	Improvement nt *Numbu*	leaf rust (R), *Anthracnose* (MR), and stem rot (HR)	69–75	61.40	0.140	9.39
UPCA S1 (V12)	56B	na	60–70	66.50	0.215	7.38

^*^ HR = highly resistant, R = resistant, MR = moderately resistant.

^**^ dap = days after planting.

^***^ na = data not available.

### 2.3 Experimental design and observation

The experiment was conducted in tidal swamplands of type C ( *Inceptisols*) and sandy soils ( *Entisols*). Intensive tillage was practiced. After one week, two seeds were planted per planting hole, with 0.6 m between rows and a 0.25 m planting distance. A replanting operation was performed 7–14 days after planting. Thinning was conducted 30 days after planting, leaving a single plant per pot. Weeds were controlled manually, with hoeing 26 and 46 days after planting.

The trials were arranged in a randomized complete block design (RCBD) with two-factor treatments and three replications. The first factor was tested environments consisted of E1 = tidal swamplands applied with 500 kg ha ^−1^ chicken manure in the wet season, E2 = tidal swamplands applied with 1000 kg ha ^−1^ chicken manure in the wet season, E3 = tidal swamplands applied with 500 kg ha ^−1^ chicken manure in the dry season, and E4 = tidal swamplands applied with 1000 kg ha ^−1^ chicken manure in the dry season, E5 = sandy soils applied with 500 kg ha ^−1^ chicken manure in the dry season, and E6 = sandy soils applied with 1000 kg ha ^−1^ chicken manure in the dry season. The second factor was 12 varieties of sorghum (  Table 1). Each plot consisted of eight 5.0-m long rows and sixteen 4.0-m long rows. The utilized area was defined as the area occupied by the central row. The entire plot was applied with 1000 kg ha ^−1^ of dolomite. The observed traits are given in  Table 2.

**Table 2.** Observed traits and codes.

Code	Trait	Measurement procedure	Measurement unit
PH	Plant Height	From the base of the stem to the top of the canopy	cm
LC	Number of Leaves	Number of leaves, including new leaf shoots	count
INC	Number of Internodes	Number of internodes	count
INL	Internodes Length	Length of space between nodes in the third or fourth internode	cm
SD	Stem Diameter	Diameter in the third or fourth internode	cm
LW	Leaf Width	The widest point across the leaf blade and the distance between the two edges at that point	cm
LL	Leaf Length	The tip of the leaf blades to the petiole	cm
PL	Panicle Length	Length from the base of the panicle to the tip of the most extended branch on the panicle	cm
SWW	Stem Wet Weight	The fresh weight of the main stem	gr
RWW	Root Wet Weight	The fresh weight of the root	gr
BRIX	sweetness level	The sweetness level at the main stem (%)	%
LWW	Leaf Wet Weight	The fresh weight of the leaf	gr
GY	Grain Yield	Clean seeds per panicle at 10% moisture content	gr

### 2.4 Data analysis

2.4.1 Analysis of variance

Multivariate Analysis of Variance for all traits according to the following model:

$$Y_{\text{ijkt}}=\mu_{t}+\beta_{k}+E_{I}+V_{j}+{\left(\mathrm{EV}\right)}_{\mathrm{ij}}+\epsilon_{\text{ijkt}}$$ [1]

Where $Y_{\text{ijkt}}\;\text{is}\;$ the observed *t*-th traits at *k*-th block under the *i*-th environment of the *j*-th variety, $\mu_{t}$ is the grand mean of the *t*-th trait, $\beta_{k}$ is the *k*-th block effect, *i* = 1, 2…e; *j* = 1, 2, 3 … v; *k* = 1, 2, 3; *t* = 1, 2 … p. $E_{i}$ is the *i*-th environmental effect, $V_{j}$ is the *j*-th variety effect, ${\left(\mathrm{EV}\right)}_{\mathrm{ij}}$ is the interaction of the variety and the environment, and $\epsilon_{\text{ijkt}}$ is the experimental error at the *t*-th trait of the *j*-th variety planted at the experimental unit under the *i*-th environment and * k*-th block. Multivariate analysis of variance (MANOVA) was conducted for *p* traits based on model (1). Based on the MANOVA results, the means of the significant effects are extracted to construct two-way tables with variety means or a combination of variety-environment means in rows and traits in columns. Let denote the two ways tables in matrices form as *V* = ( *V _jt_*) _vxp_ and *EV* = ( *EV*_( *ij*) *t*_) _evxp_, for variety means or variety-environment combination means in rows and traits *t* in column, respectively. The rows of the two matrices are then rescaled so that all columns have values 0-100 as follows. ^31, 34^

$$rV_{j(t)}=(\frac{\max_{\left(\mathrm{nt}\right)}-\min_{\left(\mathrm{nt}\right)}}{\max_{\left(\mathrm{ot}\right)}-\min_{\left(\mathrm{ot}\right)}})x(v_{j(t)}-\max_{\left(\mathrm{ot}\right)})+\max_{\left(\mathrm{nt}\right)}$$ [2]

for the column of *V*. The column of *EV* rescaled in the same way. Where $\max_{\left(\mathrm{nt}\right)}$ and $\min_{\left(\mathrm{nt}\right)}$ are the maximum and minimum values of traits *t* in column of *V* or *EV* after rescaling, respectively; $\max_{\left(\mathrm{ot}\right)}$ and $\min_{\left(\mathrm{ot}\right)}$ are the original maximum and minimum value of the trait *t*, and *Vj*( *t*) is the original value for the *t*-th trait of the *j*-th variety or variety-environment combination. The values of *max*_( *nt*)_ and *min*_( *nt*)_ determined based on whether we expect the highest value and the lowest value in the “ideotype” if we expect that the “ideotype” has the highest value for the traits then we set, $\max_{\left(\mathrm{nt}\right)}$ = 100 and $\min_{\left(\mathrm{nt}\right)}=0$ ; otherwise, $\max_{\left(\mathrm{nt}\right)}=0\;\text{and}\;\min_{\left(\mathrm{nt}\right)}$ = 100.

2.4.2 Factor analysis

The *V* ^*^ = ( *rV*_*jt*_) _vxp_ or *EV* ^*^ = ( *rV*_( *ij*) *t*_) _evxp_, i.e., the rescaled *V* and *EV* respectively are then subject to factor analysis to group variables based on their correlation. All variables within a particular group are expected to be highly correlated with one another but have relatively small correlations with variables in different groups.

The estimation of the factorial scores for each row in the two matrices ( V* and EV*) is according to the following model:

x=μ+Lf+ϵ [3]

Where ***X*** is a 1xp vector of a row of V* or EV*, μ is the 1xp vector of the standardized mean, **L** is a pxf matrix of factorial loadings, ***f*** is a px1 vector of common factors, and ϵ is a px1 vector of residuals. *p* and *f* are the number of traits and common factors retained. The initial loadings are computed considering only factors with eigenvalues of the correlation matrix of $\;{\mathrm{rV}}_{\mathrm{ij}}\;$ or ${\mathrm{rVE}}_{\mathrm{ij}}$ higher than 1. The varimax rotation criteria are used for the analytic rotation and estimation of final loadings. The scores are then obtained as follows.

$$S=X^{*}{\left(A^{T}R^{-1}\right)}^{T}$$ [4]

**S** is a vxf matrix with factorial scores, X is a vxp scaled matrix ( V* or EV*), and **A** is a pxf matrix of canonical loading. **R** is a pxp correlation matrix between the traits, and f is the number of factors retained. Factors associated with the eigenvalue of the matrix greater than one are retained.

2.4.3 Multitrait-Genotype-Ideotype-Distant Index (MGIDI)

The MGIDI _i_ for the *i-th * treatment (variety or variety-environment combination), is defined as the Euclidean distance between the scores of the *i-th * treatment and the ideal type, and computed as follows.

$$\text{MGIDI}={\left[\sum_{j=1}^{f}{\left(\gamma_{\mathrm{ij}}-\gamma_{j}\right)}^{2}\right]}^{0.5}$$ [5]

Where $\gamma_{\mathrm{ij}}\;$ is the score of the *i- *th treatment in the *j- *th factor ( *i* = 1, 2, …, *t*; *j* = 1, 2, …, *f* ), being *t* and *f* the number of treatments and the retained factors, respectively; and $\gamma_{j}$ is the *j*th score of the ideotype or ideal treatment. The treatment with the lowest MGIDI is closer to the ideal treatment, presenting the desired values for all the *p* traits. The traits are prioritized by putting the following weights (number in the bracket in front of the traits): (0.4) PH, (0.6) LC, (0.4) INC, (0.4) INL, (0.7) SD, (0.7) LW, (0.7) LL, (0.6) PL, (0.5) (PDW), (1.0) LWW, (0.3) BRIX, (1.0) SWW, (1.0) GY. The analysis was performed using *R* software version 4.3.3. ^51^

2.4.4 GGE Biplot

The mean yield of variety *i* in environment *j* according to model (1) is:

$$Y_{\mathrm{ij}}=\mu +E_{i}+V_{j}+{\left(\mathrm{EV}\right)}_{\mathrm{ij}}$$

If we delete *E*_*i*_ from *Y*_*ij*_, then the environmental-centered data matrix M with the *ij*-th element

$$m_{\mathrm{ij}}=Y_{\mathrm{ij}}-\mu -E_{i}$$

can be subjected to singular value partitioning (SVP), i.e.,

$$m_{\mathrm{ij}}=\sum_{k=1}^{p}a_{\mathrm{ik}}\lambda_{k}^{f}\lambda_{k}^{1-f}b_{\mathrm{jk}}$$

Where $\xi_{\mathrm{ik}}=a_{\mathrm{ik}}\lambda_{k}^{f}\;$ and $\lambda_{k}^{1-f}b_{\mathrm{jk}}$ ; are the PC score for variety *i* and environment *j*, respectively; *f* is the single value partition factor for the Principal Component (PC) and $\lambda_{k}\;$ is the *k*-th eigenvalue of matrix M. In this research the biplots are constructed at *f *= 0 (SVP = 2), meaning that singular value is entirely partitioned into environment eigenvectors.

A Genetic plus Genetic-Environment interaction (GGE) biplot was used to examine the stability and adaptability of the varieties. The biplot’s abscissa represents the first principal component (PC1), indicating the phenotypic performance of the varieties, while the ordinate represents the second principal component (PC2), indicating the stability of the varieties. The two components account for the variation in varieties and the interaction between varieties and environments. By connecting the variety’s coordinates that were most distant from the origin, a polygon was created that can be used to determine which varieties were the best or worst and at which environments (  Figure 5 and Figure 10). The biplot is divided into sectors by drawing a dotted line perpendicular to the polygon’s sides from the origin of the biplot. The sectors depict environments that are most comparable to one another. The varieties with the best or the worst phenotypic performance in environments within a sector were those found near the polygon’s vertices in the sector. A group of environments where the same variety performs the best is called a mega-environment. Varieties in a sector without allocated environments are considered unfavourable to any environment and exhibit low phenotypic performance responsiveness.

**Figure 1.**
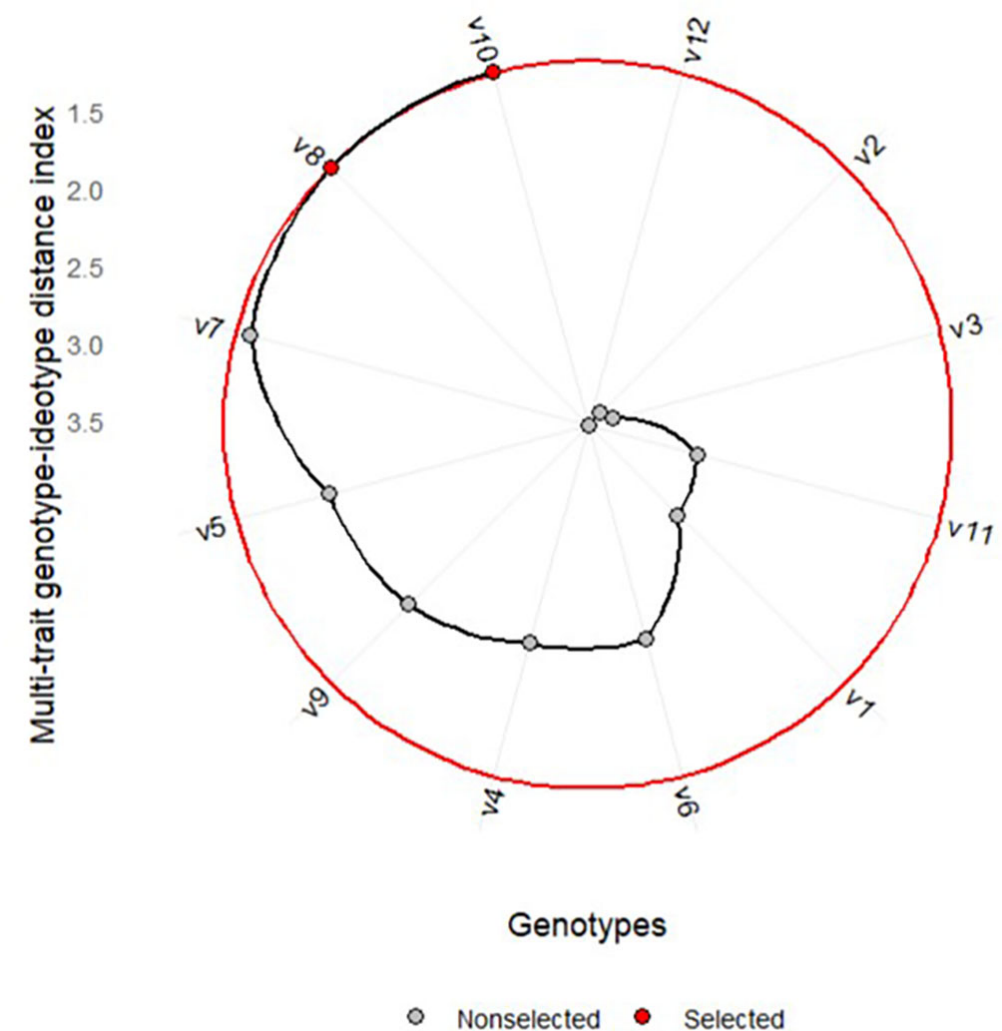
Variety ranking based on MGIDI.

**Figure 2.**
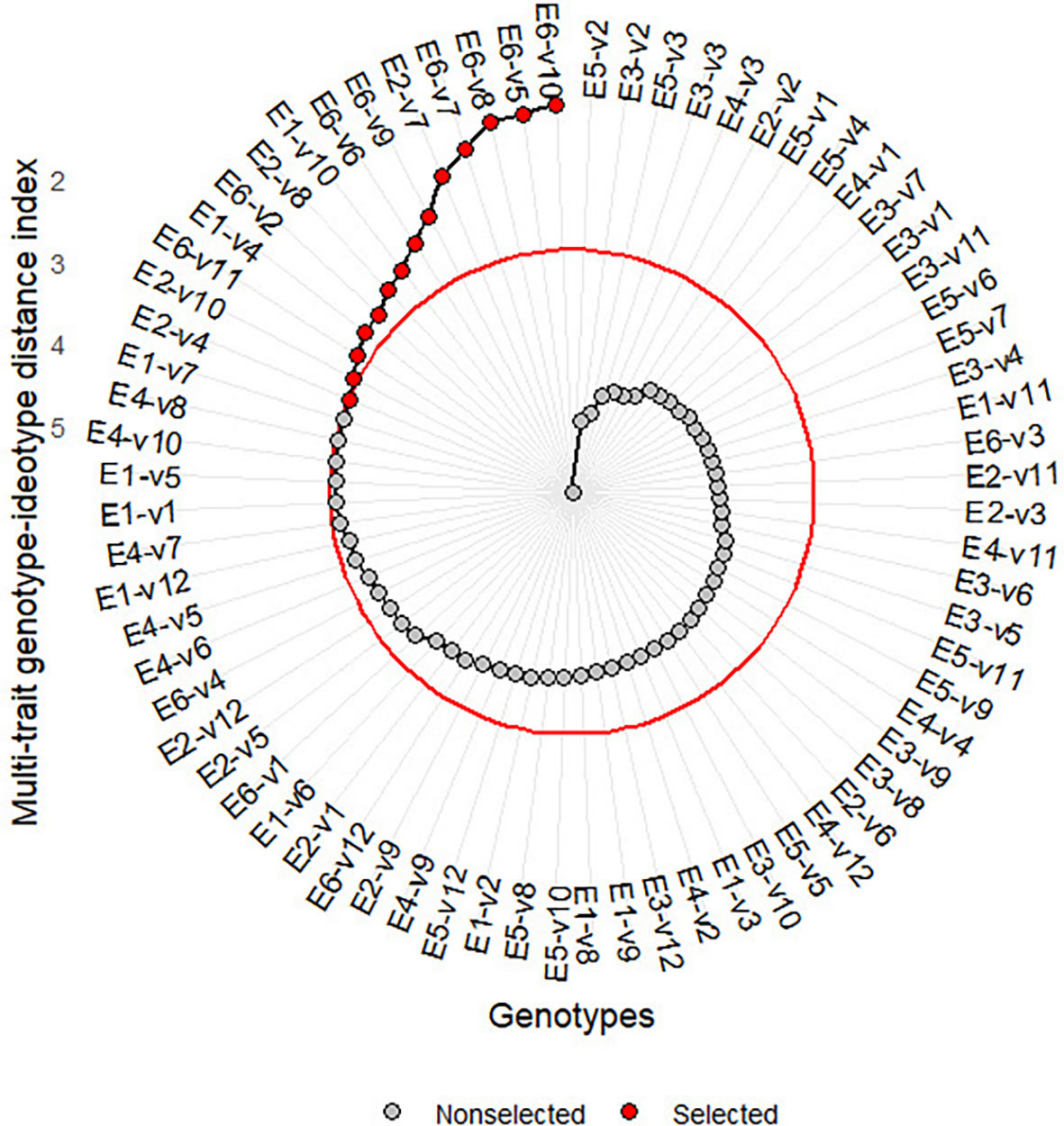
Variety-environment ranking based on MGIDI.

**Figure 3.**
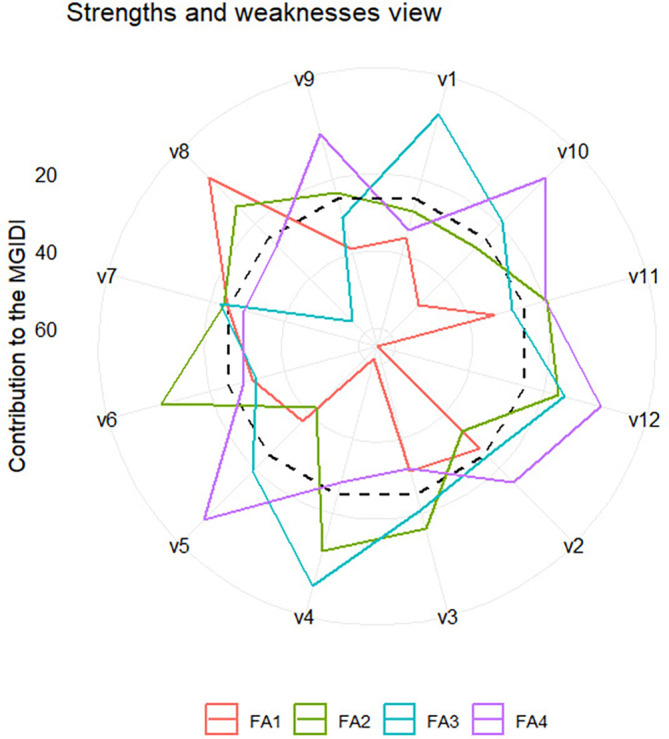
Strength and weakness view of all varieties.

**Figure 4.**
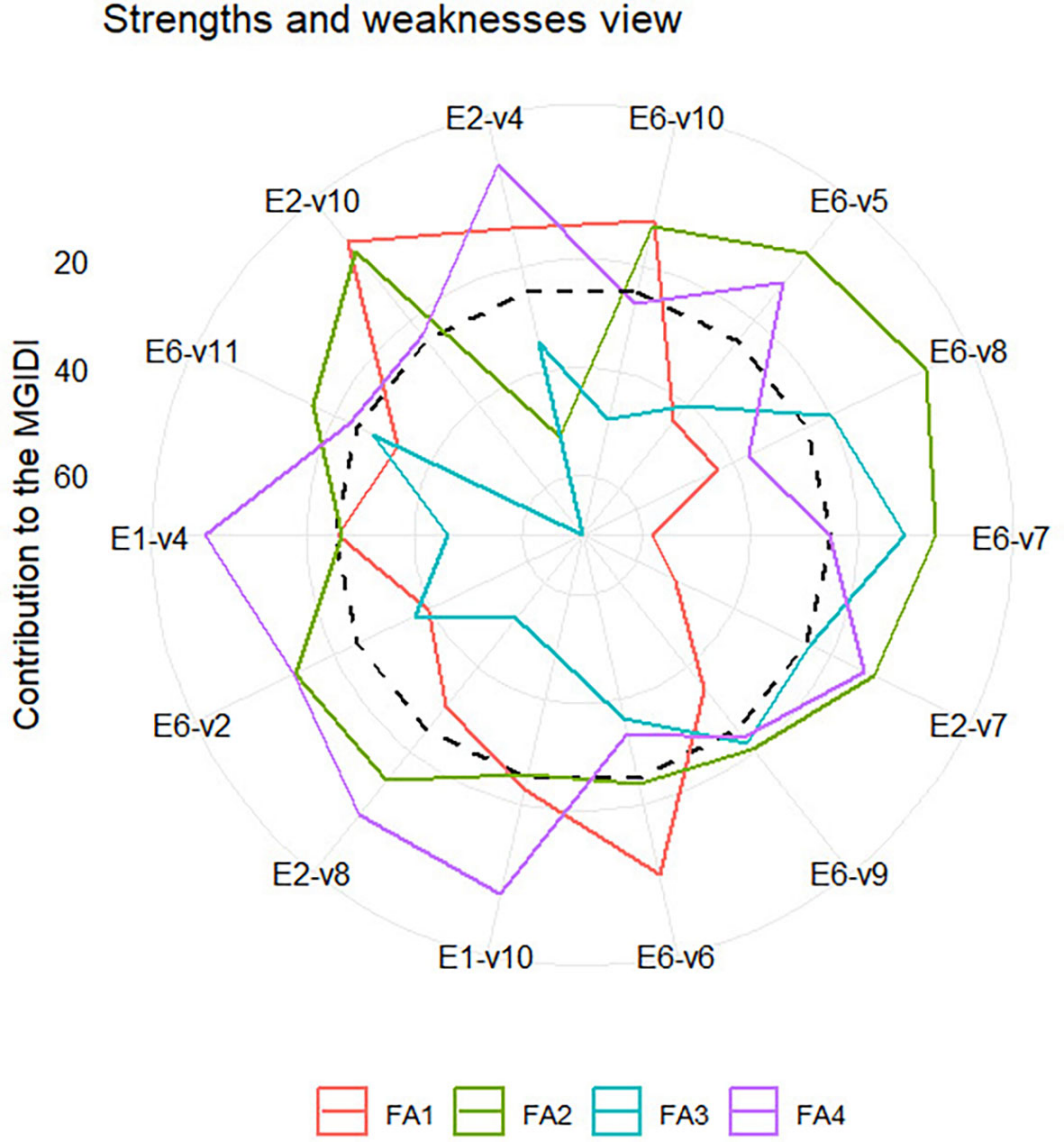
Strength and weakness view of selected varieties - environmental combination.

**Figure 5.**
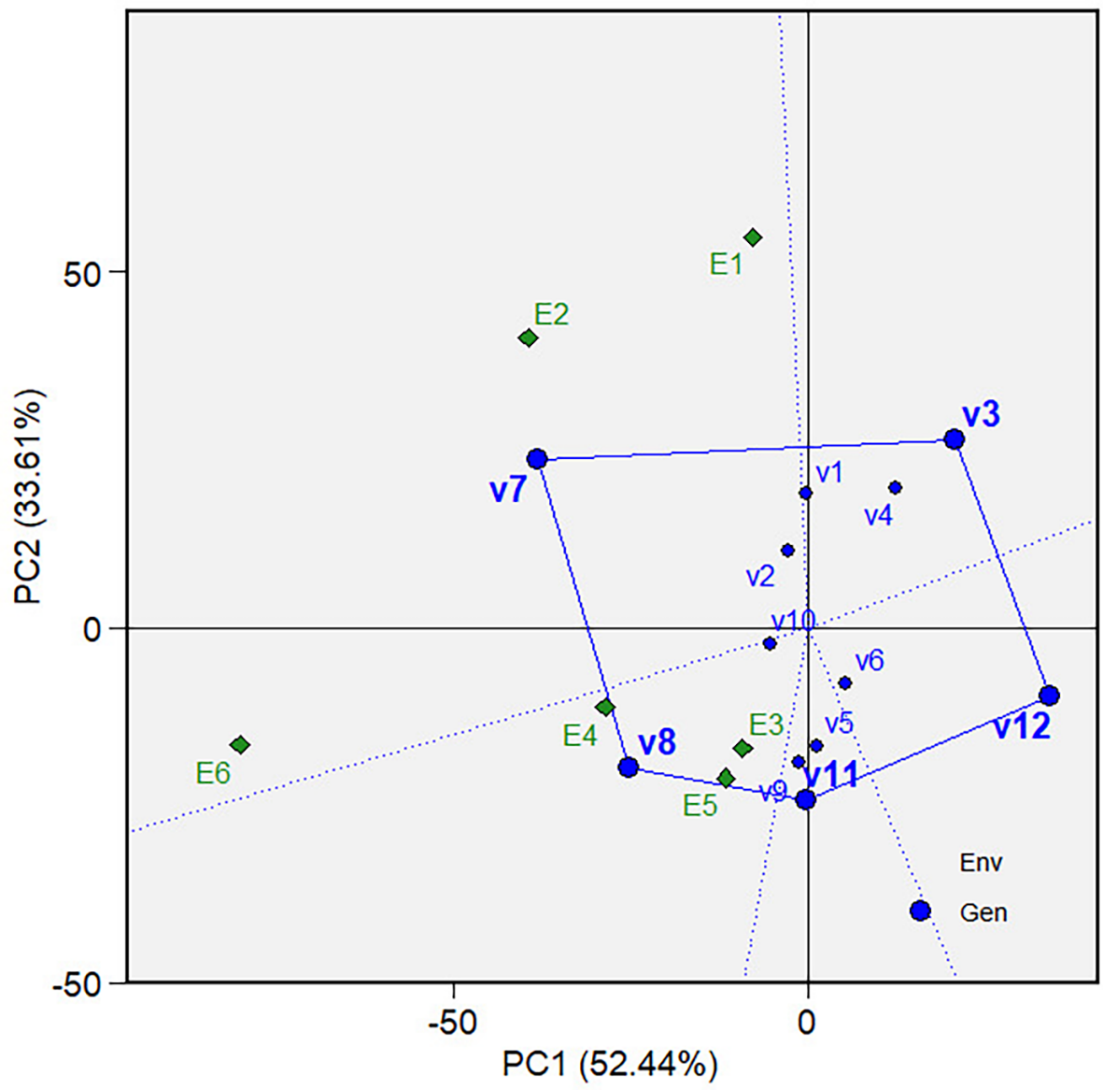
Which-won-where view of the GGE biplot on grain yield.

**Figure 6.**
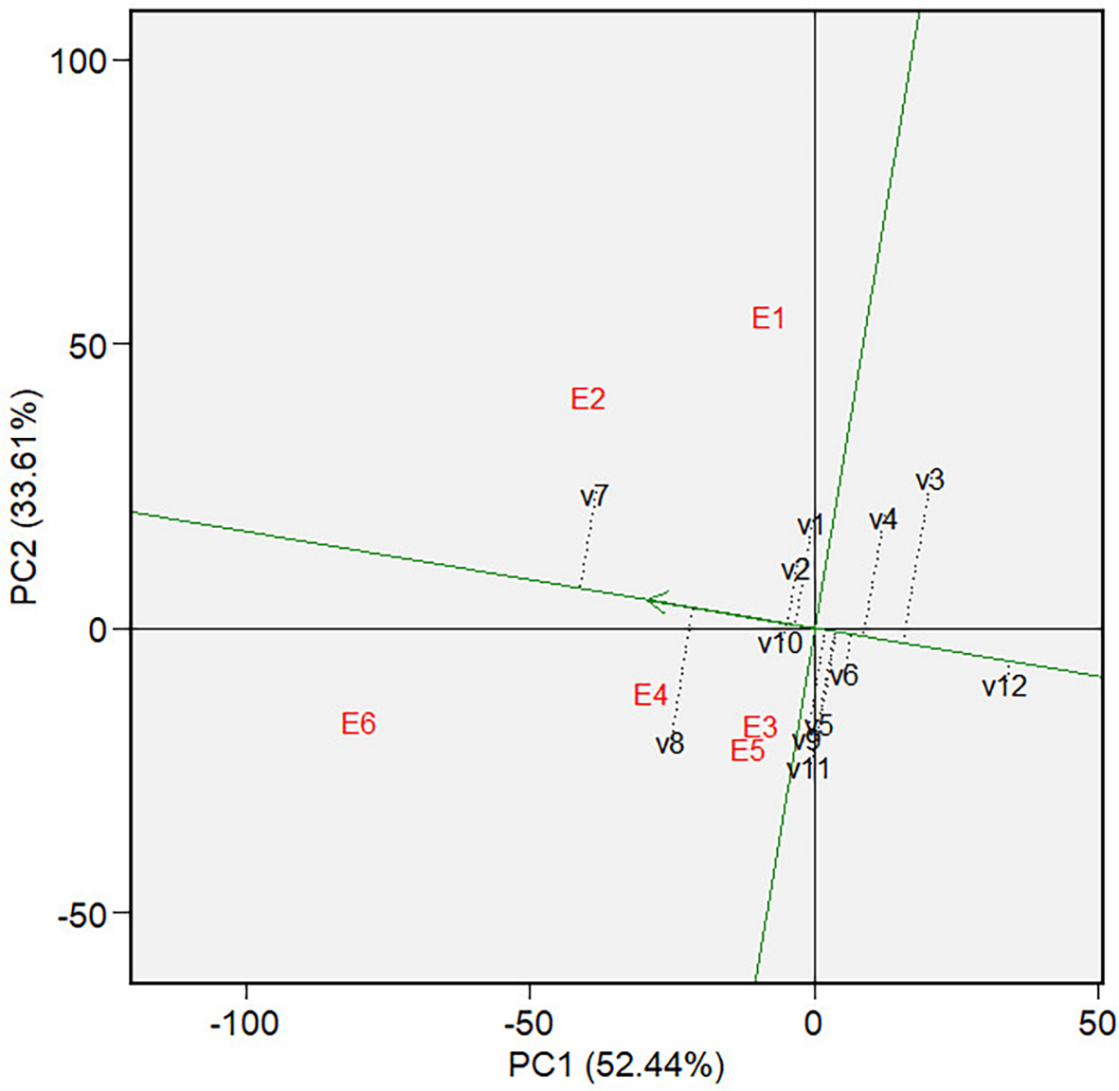
Mean vs. stability of varieties GGE biplot on grain yield.

**Figure 7.**
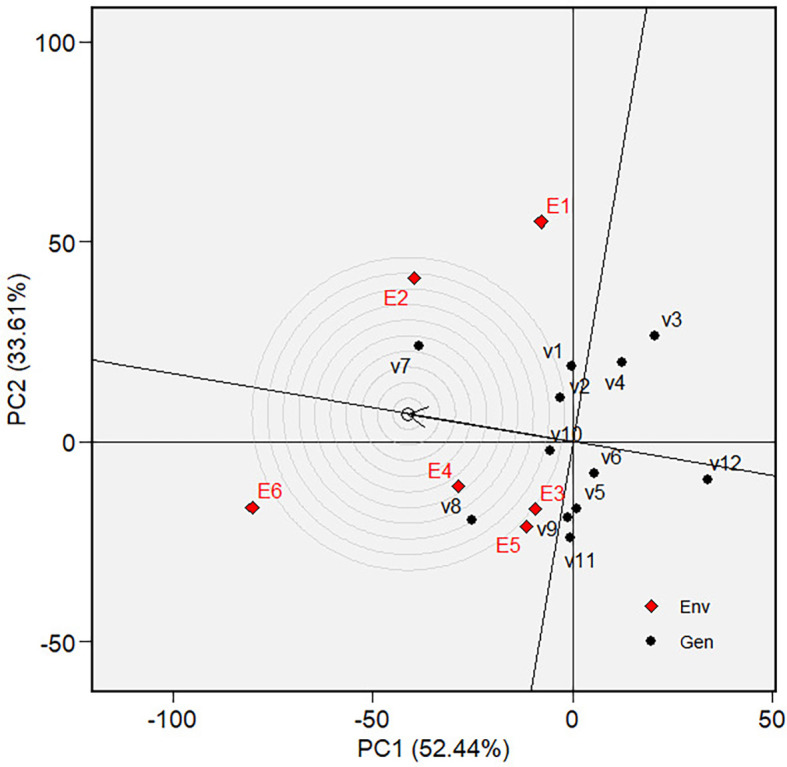
Ranking of varieties in GGE biplot on grain yield.

**Figure 8.**
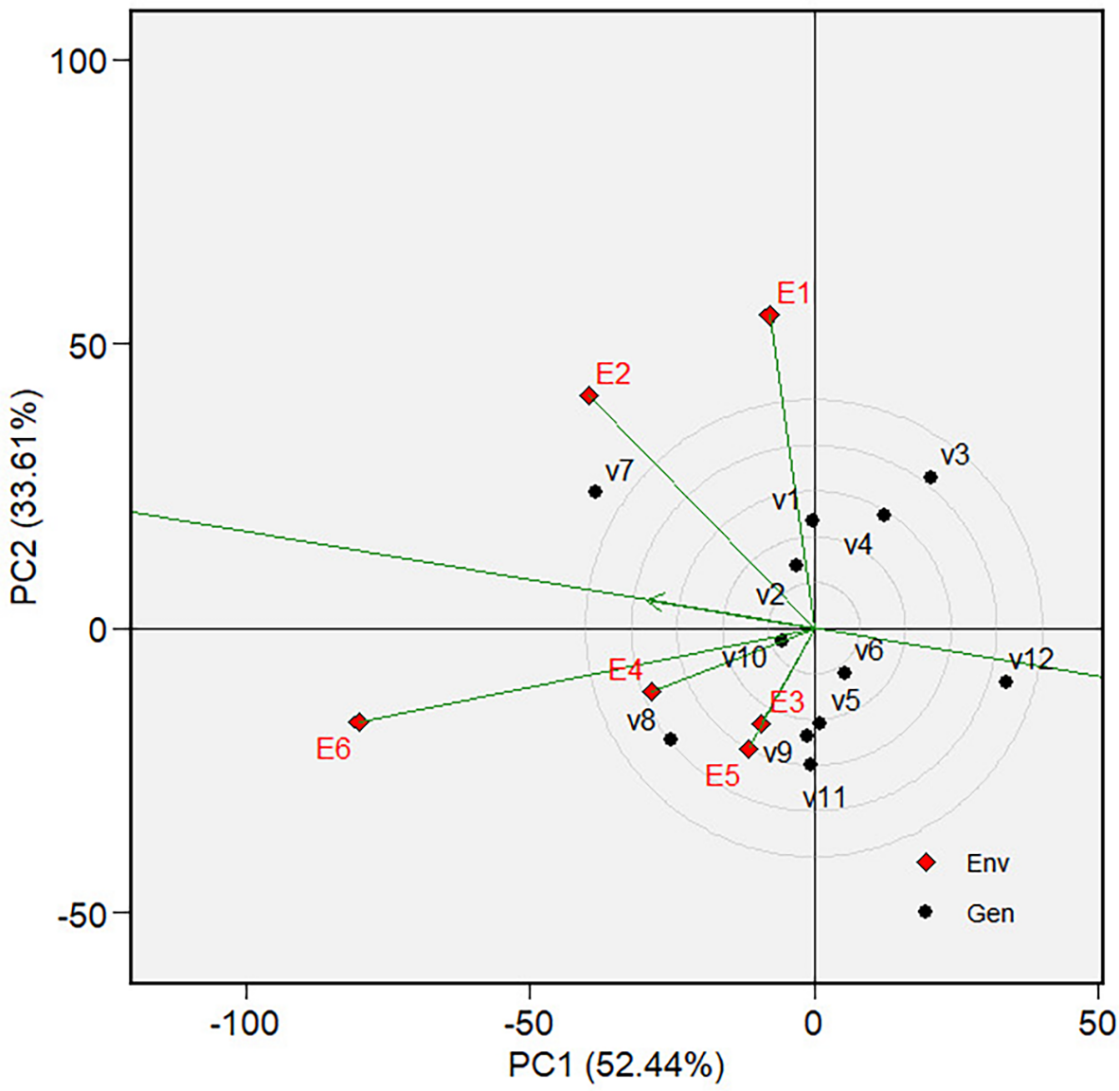
Discriminativeness and representativeness of the tested environments in the GGE biplot on grain yield.

**Figure 9.**
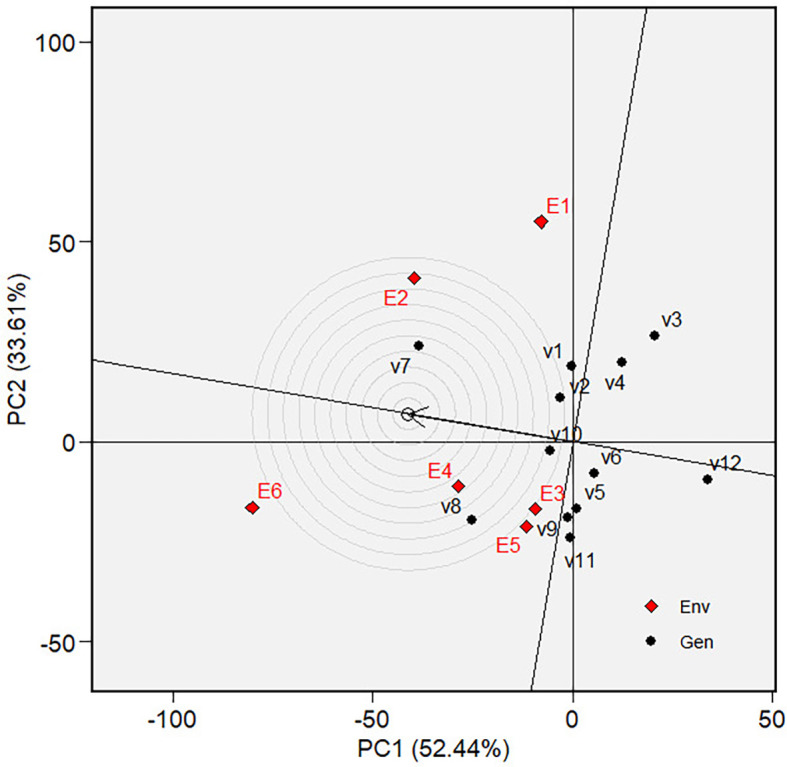
Ranking tested the environments of the GGE biplot on grain yield.

**Figure 10.**
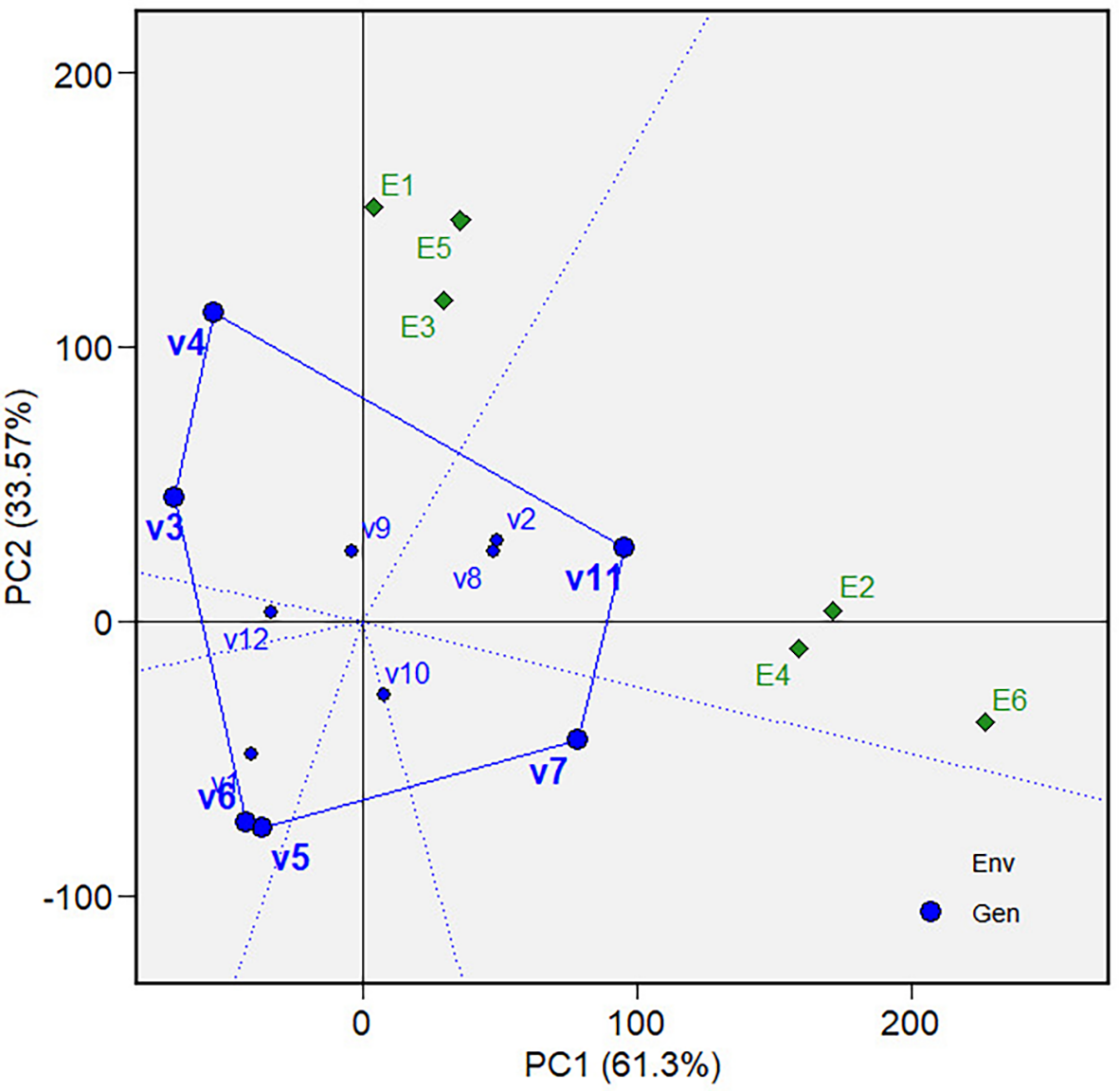
The Which-won-where View of the GGE biplot based on the forage wet weight.

The average environmental point, with coordinates representing the average PC1 and PC2 scores of the environments, was initially defined to create the Average Environmental Coordination (AEC). The AEC’s X-axis is a line between the biplot’s origin and the average environmental point. Simultaneously, the Y-axis is the line that runs perpendicular to the AEC’s X-axis in the biplot’s origin. The ordinate shows the interactions between each variety and its environment, whilst the AEC abscissa shows the phenotypic performance of varieties in the average environment. The arrow in the AEC axis indicates the direction of ascending phenotypic performance. The projection of each variety on the X-axis of AEC measures the mean phenotypic performance across environments.

**Figure 11.**
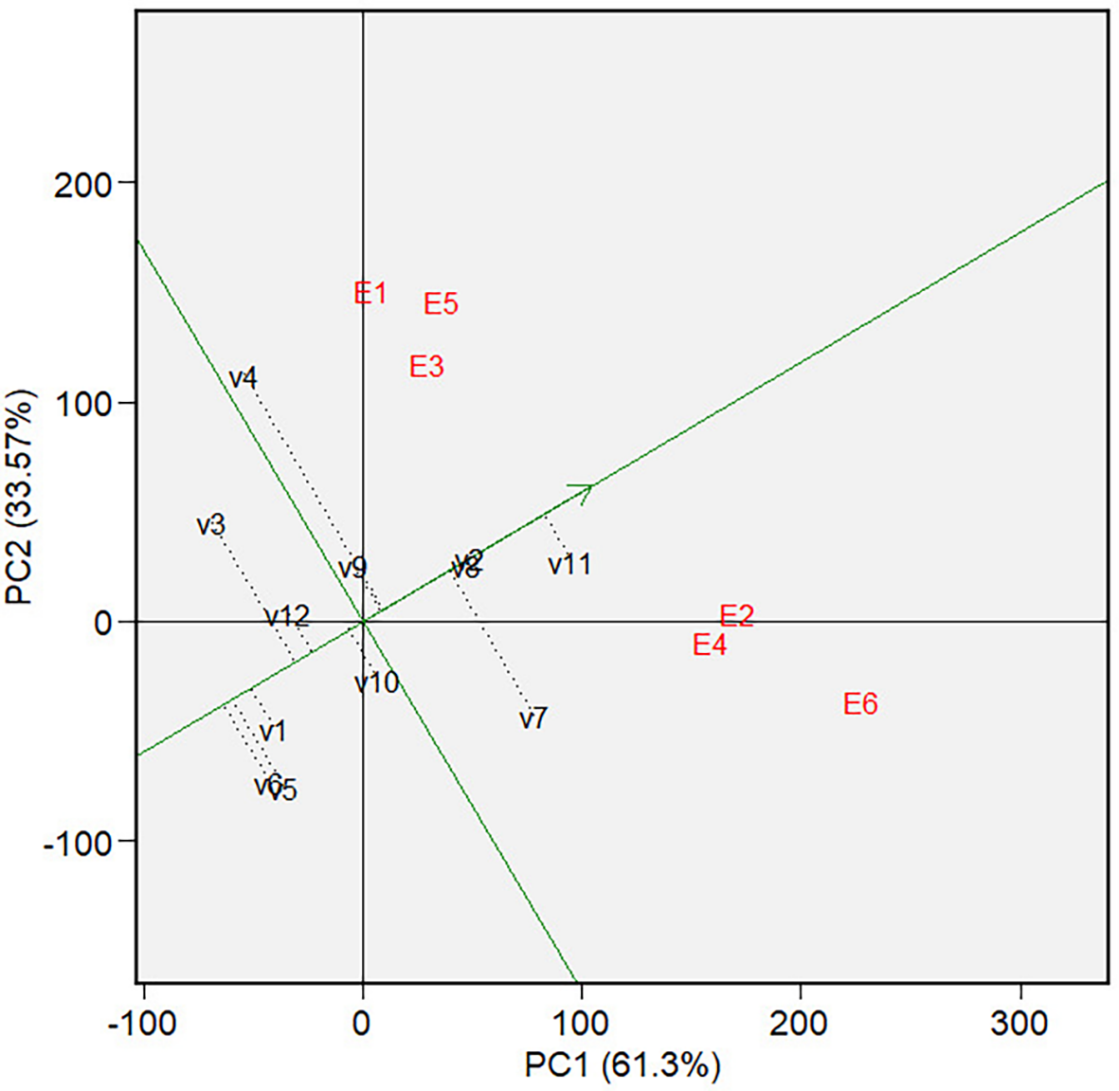
Mean and stability of varieties at GGE biplot on forage yield.

**Figure 12.**
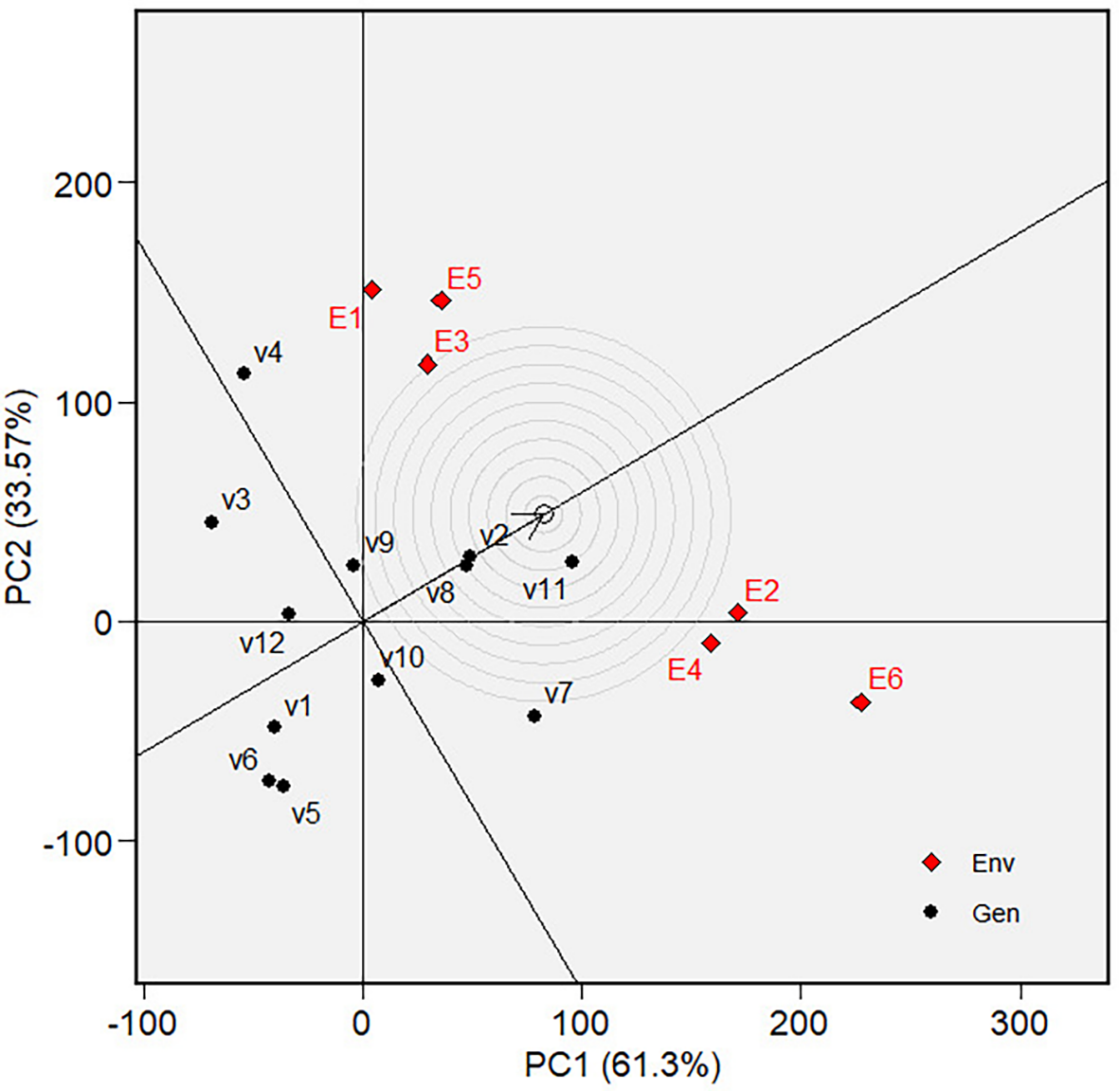
Ranking of varieties at GGE biplot on forage yield.

In contrast, the projection on the Y-axis measures the stability of the variety in tested environments (  Figure 6,  Figure 11). The ascending direction is the arrow in the abscissa, and the varieties projected above the origin in the direction of the arrow in the abscissa are above the average of the mean phenotypic performance; the higher the ordinate of the variety in the AEC coordinate is, the less stable it is. The best (adaptable) variety is the highest phenotypic performance and stability variety. This imaginary “ideal variety,” i.e., the best variety, is marked as a small circle in  Figures 7 and 12. Varieties are ranked by their mean phenotypic performance and stability, as indicated by their closeness to the “ideal variety” (  Figures 7 and 12).

**Figure 13.**
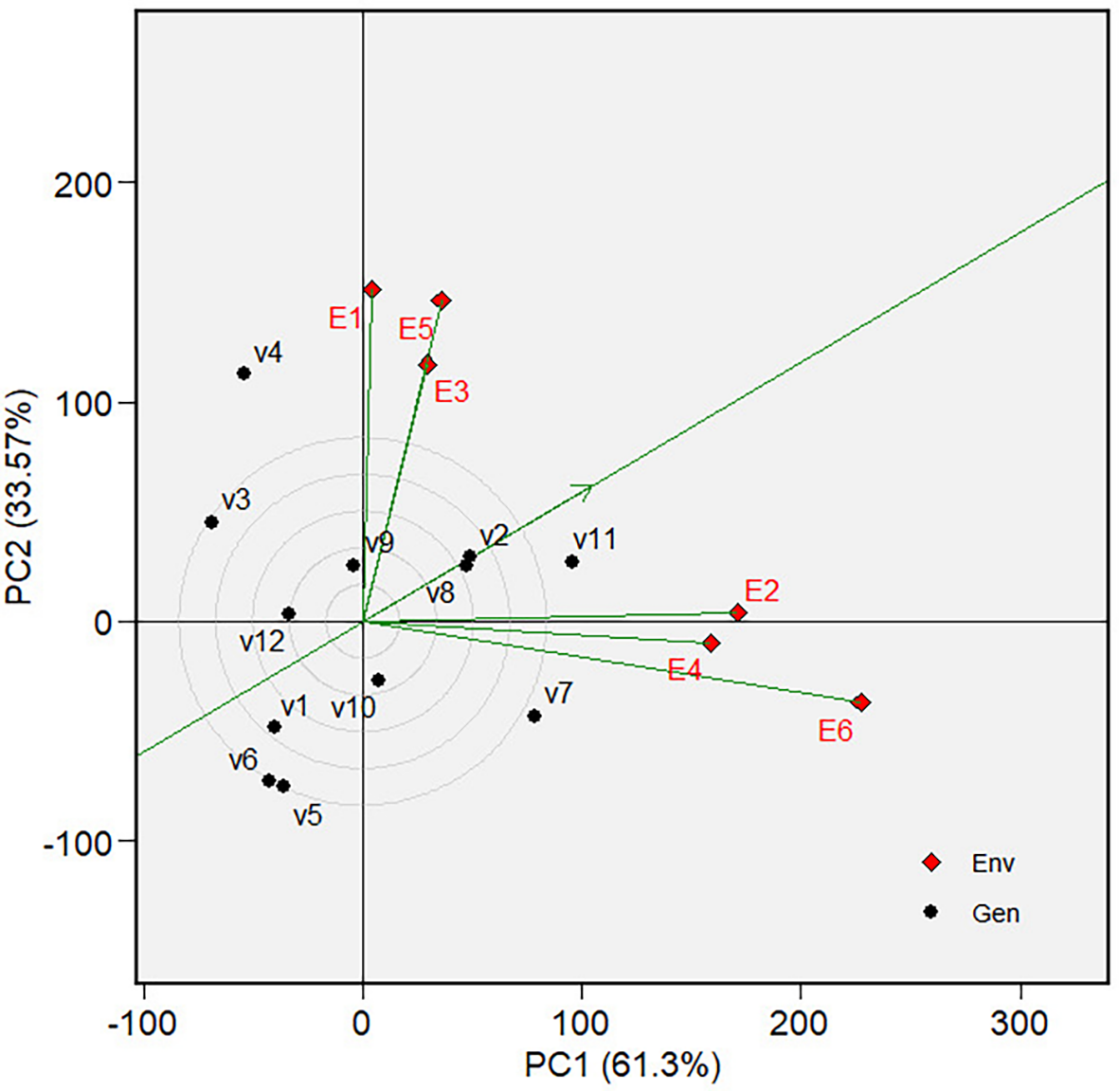
Discriminativeness and representativeness of environments of the biplot on forage yield.

**Figure 14.**
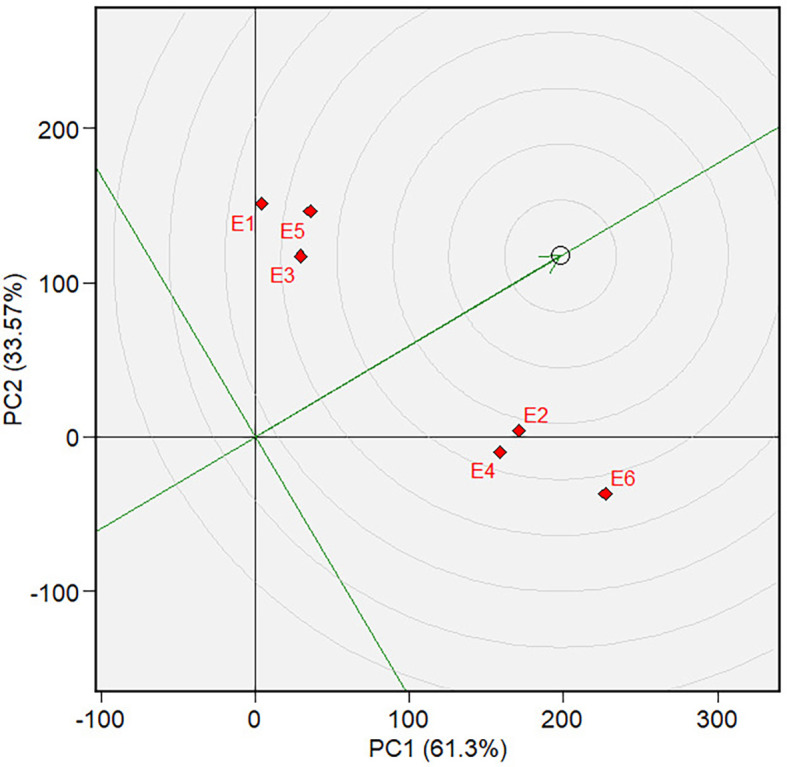
Ranking of environments of the biplot on forage yield.

The ideal variety is based on its performance in the AEC. However, one may need to determine a test environment representing the average environment. A line vector was constructed from the biplot’s origin to each environmental point to evaluate the environment’s representativeness and discriminating power. The length of the vector represents the discriminating ability of the environment, while the angle between the vector and the X-axis of AEC measures the representativeness of the environment. The longer the vector and the smaller the angle, the higher the discriminating ability and representativeness of the environment associated with the vector (  Figures 8 and 13). The environment is then ranked based on its discriminativeness and representativeness (  Figure 9 and Figure 14).

## 3. Result

### 3.1 Analysis of variance

The multivariate analysis of variance (  Table 3) found that variety means across environment ( *V*) and variety-environment interaction ( *VE*) have significant effects on the vector of traits, based on the *Pillai* trace Test ( *p *< 0.01), indicating differences in the means of varieties across environments and such differences are affected by environment. The significant effect of variety-environment interaction means that the ranking of varieties within each environment is varied.

**Table 3.** Multivariate analysis of variance on traits.

Source	Df	*Pillai*	Approx F	num Df	den Df	Pr(>F)
Rep.	2	0.4987	3.3472	26	262	1.265 e-06 ^***^
Env.	5	3.2940	19.9033	65	670	<2.2 e-16 ^***^
Var.	11	5.5343	10.9043	143	1540	<2.2 e-16 ^***^
Env:Var	55	5.0732	1.6524	715	1846	<2.2 e-16 ^***^

Note: Significance. codes: 0 ' ^***^’ 0.001 ‘ ^**^’ 0.01 ‘ ^*^’ 0.05 ‘.’ 0.1 “1

### 3.2 Factor analysis

Two two-way tables were extracted from the MANOVA: *V* = ( *V _ij_*) _vxp_, i.,e., the rows are varieties and the columns are the traits, and *EV* = ( *EV*_( *ij*) *t*_) _(ev)xp_, i.e., the rows are the variety-environment combinations and the columns are the traits. Factorial loading after varimax rotation and their cumulative variance obtained in factor analysis on the variety mean matrix (V) are presented in  Table 4. In contrast, the variety-environment combinations matrix (EV) is presented in  Table 5. In both tables, four factors associated with an eigenvalue greater than one are retained along with their cumulative variance. The bold-faced numbers (greater than 0.50 in absolute value) in each table are the dominant factor loading of the traits to the associated factor. Hence, for example, in Table 4, internode count (INC), panicle dry weight (PDW), stem wet weight (SWW), and grain yield (GY) are associated with factor 1(FA1). Similarly, plant height (PH), Internode count (INC), internode length (INL), leaf length (LL), and BRIX are associated with the factor (FA2). Factor 3 is associated with panicle length (PL),) and BRIX. Factor 4 is associated with leaf count (LC), stem diameter (SD), leaf width (LW), panicle dry weight (PDW), and Leaf wet weight (LWW). Similar interpretations can also be held for  Table 5. The result of factor analysis will then be used to calculate MGIDI.

**Table 4.** Factorial loadings explained variance and eigenvalues after varimax rotation obtained in factor analysis on variety—means matrix.

Traits		Factors		
	FA1	FA2	FA3	FA4
PH	-0.28	- **0.88**	-0.02	0.37
LC	-0.05	-0.04	-0.09	**0.83**
INC	**-0.57**	**-0.62**	0.1	0.44
INL	-0.22	**-0.91**	0.02	0.01
SD	-0.49	-0.17	0.03	**0.74**
LW	-0.45	-0.2	-0.25	**0.69**
LL	-0.45	**-0.71**	-0.38	-0.34
PL	0.26	-0.02	**-0.87**	0.21
PDW	**-0.69**	0.03	0.28	**0.62**
LWW	0.04	-0.46	0.33	**0.69**
BRIX	0.05	**-0.55**	**0.6**	-0.02
SWW	**-0.92**	-0.28	0.13	0.16
GY	**-0.92**	-0.32	0.12	0.09
Eugenvalue	6.91	1.85	1.29	1.25
Cumulative variance	58.10%	67.40%	77.30%	86.90 **%**

Note: The numbers in bold are the high loading of the traits on the respective factors (column).

**Table 5.** Factorial loadings explained variance and eigenvalues after varimax rotation obtained in factor analysis on variety—environment combinations mean matrix.

Traits		Factors		
	FA1	FA2	FA3	FA4
PH	**-0.84**	0.35	0.09	0.02
LC	0.01	**0.84**	0.04	0.03
INC	**-0.77**	0.28	0.02	0.05
INL	**-0.82**	0.03	0.16	-0.04
SD	-0.47	0.11	0.36	**-0.69**
LW	-0.47	0.37	0.35	**-0.62**
LL	**-0.74**	0.25	0.39	**-**0.36
PL	0.09	0.15	0	**-0.86**
PDW	-0.09	**0.71**	**0.52**	0.02
LWW	-0.43	**0.62**	0.09	-0.04
BRIX	**-0.58**	0.21	-0.43	-0.16
SWW	-0.15	0.14	**0.95**	-0.10
GY	-0.19	**0.1**	**0.94**	-0.15
Eugenvalue	**5.7**	**1.74**	**1.66**	**1.04**
Cumulative variance	**43.80** *%*	**57.3** *%*	**70.00** *%*	**78.00%**

Note: The numbers in bold are the high loading of the traits on the respective factors (column).

### 3.3 Selection based on MGIDI

3.3.1 Selected varieties

Figure 1 shows the ranking of the MGIDI of varieties averaged across environments. The selected varieties based on the MGIDI are Kawali (V10) and *Numbu* (V8), as indicated by the red dots in  Figure 1.

3.3.2 Selected variety-environment combinations

Figure 2 presents the ranking of variety-environment combinations based on MGIDI. The red dot at the outer circle is the selected environment-variety combination. They are E6-V10, E6-V5, E6-V8, E6-V7, E2-V7, E6-V9, E6-V6, E2-V8, E1-V4, and E1-V10, E6_V11, E2_V10, E2_V4, E6_V2, where *E _i_V _j_* denotes the *j-th * variety planted at the *i-th * environment. Most selected varieties are those applied in sandy soil with a high rate of organic fertilizer (E6). Only four varieties (V4, V7, V8, or V10) were selected for tidal swamplands in the rainy season, and they were either applied at a high or low rate of organic fertilizer (E1 and E2).

3.3.3 Selected varieties in each environment

The multivariate analysis of variance, as shown in  Table 3, indicates that the interaction between variety and environment is significant. This interaction suggests that the ranking of varieties varies across different environments. Therefore, it is necessary to select varieties in each environment by the MGIDI. The selection procedure is similar to that of average varieties across all environments and variety-environment combinations. However, the result of the factor analysis and the graphs of the ranking are not presented here.  Table 6 presents the result of the selection.

**Table 6.** Selected varieties in each environment.

Environment	Selected varieties
500 kg ha ^−1^ chicken manure applied in tidal swampland in the wet season (E1)	*Suri 1 Agritan* (V1) and *Soper 4 Agritan* (V4)
1000 kg ha ^−1^ chicken manure applied in tidal swampland in the wet season(E2)	*Soper 7 Agritan* (V7) and *Numbu* (V8)
500 kg ha ^−1^ chicken manure applied in tidal swampland in the dry season (E3)	*Soper 7 Agritan* (V7) and *Bioguma II Agritan* (V11)
1000 kg ha ^−1^ chicken manure applied in tidal swampland in the dry season (E4)	*Numbu* (V8) and *Kawali* (V10)
500 kg ha ^−1^ chicken manure applied in sandy soils in the dry season (E5)	*Numbu (V8)* and Kawali (V10)
1000 kg ha ^−1^ chicken manure applied in sandy soils in the dry season (E6)	*Numbu (V8)* and Kawali (V10)

### 3.4 The strengths and weaknesses view

The strengths and weaknesses of all varieties and selected varieties-environment combinations, which are accounted for by the proportion of each factor to their calculated MGIDI, are presented in  Figure 3 and  Figure 4, respectively. Each factor has a specific color line, as indicated by the legend. The closer the variety or variety-environment combinations are to the external edge of the polygon, with a specific color representing a particular factor, the smaller the contribution of the factor to the MGIDI. The smaller the contribution of a factor to the MGIDI of a variety/variety-environment combination, the closer the traits associated with the factor to the “ideal type.” Since we defined “ideal type” as those varieties or variety-environment combinations with the highest values in all traits (as selection goals), it implies that the traits associated with the factor are high in the varieties or variety-environment combinations.

The strengths and weaknesses of all varieties are shown in  Figure 3. We should focus attention on the selected varieties, i.e., V8 and V10. Variety V8 is closely related to FA1 and V10 is closely related to FA4. It implies that FA1 has a small contribution to the MGDI of V8, and hence traits like internode count (INC), panicle dry weight (PDW), stem wet weight (weight (SWW), and grain yield (GY), which are associated with FA1, have high values in V8. Similarly, V10 exhibits high values in traits related to FA4, including leaf count (LC), stem diameter (SD), panicle dry weight (PDW), and leaf wet weight (LWW).

Figure 4 illustrates the strengths and weaknesses of selected variety-environment combinations. Unlike  Figure 3, Figure 4 presents only the selected variety-environment combinations for simplicity, given the high number of variety-environment combinations. Factor FA1 makes a small contribution to the MGIDI of E1-V10, E6-V10, and E6-V6, indicating that traits associated with this factor in that variety-environment combination are similar to those in the variety-environment idiotype. Therefore, traits such as plant height (PH), internode count (INC), Internode Length (INL), leaf Length (LL), and BRIX have high values in that variety-environment combination. With similar reasoning traits associated with FA2, such as leaf count (LC) and panicle dry weight (PDW), these values must be high in variety-environment combinations E6-V8, E6-V7, E2-V7, E6-V5, and E6-V11. Two economically valuable traits, grain yield (GY) and stem wet weight (SWW), contribute to biomass production associated with FA3. This factor has made a small contribution to the MGIDI of E6-V7, E2-V7, E6-V9, and E6-V8. Therefore, these varieties must possess high values for both traits. Finally, traits that are associated with FA4 must have high values in E1_V4, E1_V10, E2-V4, E6_V2 and E2_V8.

### 3.5 Adaptability and stability

The adaptability and stability of each variety were studied, with valuable traits including grain yield and fresh forage yield (stems and leaves, expressed as wet weight). The GGE biplot on each of the two traits was used to study the adaptability and stability of varieties. The mean of varieties across environments of the two traits and their confidence intervals is presented in Table 7.

**Table 7.** Grain yield and forage yield mean of varieties across environments.

Varieties	Mean (gr/plot)		95 % Confidence interval (grain yield)		95% Confidence interval (forage yield)	
	Grain yield	Forage yield	Lower	Upper	Lower	Upper
V1	33.8	92.8	27.9	39.7	65.4	120
V2	35.5	170.8	29.7	41.4	43.4	198
V3	29.02	108.6	23.1	34.9	81.2	136
V4	29.8	139.4	23.9	35.7	112.0	167
V5	29.0	86.3	23.1	34.9	58.9	114
V6	29.3	83.6	23.4	35.2	56.2	111
V7	54.2	162.6	48.3	60.1	135.2	190
V8	45.4	167.8	39.5	51.3	140.4	195
V9	34.2	138.8	28.3	40.1	114.4	166
V10	37.1	127.9	31.2	43.0	100.5	155.9
V11	34.5	196.0	28.6	40.4	168.7	223
V12	17.1	115.1	11.3	23.0	187.7	142

3.5.1 Grain yield

The “Which-won-where view” of the biplot on the grain yield (GY) and its polygon is displayed in  Figure 5. Of the total GGE variation, the PC1 and PC2 contributed 52.44% and 33.61%, respectively. PC1 reflects the average performance (mean grain yield) of the varieties, while PC2 reflects the stability (variety-environment interaction) of the varieties/genotypes. Jointly, the two components account for 86.05% of the total genotype plus genotype × environment interaction. The polygon separated the biplot’s five sectors. The highest or the lowest phenotypic performance (mean grain yield) varieties were the varieties at the vertices of the polygon. There are five varieties at the polygon’s vertices, i.e., V3, V7, V8, V11 and V12. These varieties are candidates for the best adaptable varieties. There are two mega-environments in the biplot. Mega-environment 1 consists of environments E1, E2, and E6 in one sector, with a variety at the vertex, V7, while mega-environment 2 consists of environments E3, E4, and E5, with a variety at the vertex, V8. Varieties V3, V11, and V12 were found in sectors with no allocated environment. Hence, they were less responsive and exhibited low phenotypic performance (in terms of grain yield) in all tested environments.

The mean and stability analysis depicted in  Figure 6 shows that V7 has the highest mean in Mega-Environment 1, as it is the furthest left along the green AEC line. Note that the green AEC arrow points to the left; therefore, varieties further in that direction can be interpreted as having a higher mean performance (in grain yield). V8 is the second-highest performance, with similar reasoning. Additionally, a contrast comparison test ( Table 8) showed that V7 differs significantly from the average grain yield of other varieties. In terms of stability, which is reflected by the ordinates in AEC, V7, and V8 are moderately stable, although they are less stable than other varieties, such as V1, V2, and V10. The ranking of varieties based on their mean performance (in terms of grain yield) and stability is presented in  Figure 7. The best varieties, which are the most adaptable, are those closest to the ideal variety (represented by the small circle near the arrow), an imaginary genotype or variety with the highest mean grain yield and stability. V7 is the most adaptable variety, followed by V8. Consequently, in mega-environment 1, i.e., tidal swamplands in rainy season applied with high rate (E2) or low rate organic fertilizer (E1), and in sandy soils applied with high rate organic fertilizer (E6), the adaptable variety is *Soper 7 agritan (V7*), while in tidal swampland at dry season applied with high rate (E4) or low rate organic fertilizer (E3) and in sandy soils applied with low rate of organic fertilizer (E5) the adaptable variety is *Numbu* (V8).

**Table 8.** Contrast comparison test in mean grain yield among varieties.

contrast	estimate	SE	df	lower.CL	upper.CL	t.ratio	p.value
V7 vs others in E1	10.1	8.39	142	-6.477	26.7	1.205	0.2303
V7 vs others in E2	17.3	8.39	142	0.752	33.9	2.066	0.0406
V7 vs others in E6	17.6	8.39	142	1.044	34.2	2.101	0.0374
V8 vs others in E3	25.0	8.39	142	8.401	41.6	2.978	0.0034
V8 vs others in E4	19.1	8.39	142	2.529	35.7	2.278	0.0242
V8 vs others in E5	28.8	8.39	142	12.190	45.4	3.430	0.0008

Since the “ideal variety” in the environmental average is only hypothetical, we may need to determine the phenotypic performance of the varieties in a particular tested environment that represents the average environment. For this purpose, we first determined the tested environment that was more discriminative and representative of the average environment. The discriminativeness and representativeness of all tested environments were analysed in  Figure 8. The highest line vector from the origin of the biplot to the environment “point” was the most discriminative environment. At the same time, the most representative is the line vector with the lowest angle to the average environment. The selected environments are ranked based on their discriminativeness and representativeness **(** Figure 9). The center of the concentric circles in  Figure 9 represents the ideal environment for selecting genotypes, i.e., the most discriminative and representative ones. The closer an environment is to this center, the better it ranks. Hence, E6 is the most discriminative and representative environment of the average environment. In other words, sandy soil applied with a high rate of organic fertilizer during the dry season (E6) is ideal for selecting broadly adaptive genotypes or varieties based on grain yield (GY).

3.5.2 Forage yield

Figure 10 depicts a biplot of sorghum varieties’ Forage yield (FY) and its polygon. PC1 and PC2 contributed 61.30% and 33.57%, respectively, and jointly accounted for 94.87% of the overall GGE variance. There are two mega-environments in the biplot. The first mega-environment is in the sector that contains E1, E3, and E5 tested environments, and the second mega-environment is in the sector that contains E2, E4, and E6 tested environments. We can define the first mega-environment as the environment applied with a low rate of organic fertilizer since all environments are those applied with a low rate of organic fertilizer (500 kg of chicken manure per hectare) in both types of land at both seasons.

For the same reason, we can define the second environment as the one applied with a high rate of organic fertilizer (1,000 kg of chicken manure per hectare). Varieties V3 and V4 are at the vertices of polygons within mega-environment one and become the candidates for the best varieties in the environment. Variety V11 is the candidate for the best varieties in mega-environment 2. Varieties V5, V6, and V7 were found in sectors with no environmental conditions, indicating that they are not responsive and exhibit low mean phenotypic performance in any tested environment.

The graph of mean and stability (  Figure 11) showed that among the three varieties in mega-environment 1, V3 has a phenotypic performance (mean forage yield) below the average, while varieties V4 and V9 are above the average, with almost similar phenotypic performance. In contrast comparison test ( Table 9), V4 and V9 were not statistically different in environments E1, E2, and E6, whereas V3 and V4 were considerably different in environments E2 and E6, with the exception of environment E1. This result may explain why, although V9 is not at the vertex of the polygon. It has greater mean forage yield than V3, and has the same mean forage yield as V4. In genotype rank ( Figure 12), V9 is closer to the “ideal variety” than V3 or V4 in this mega-environment. However, it is further from the ‘ideal variety’ than V11, V2, and V8, which are in mega-environment 2. Therefore, we can conclude that in mega-environment 1, i.e., the environment in tidal swampland applied with low (E1) or high rate (E2) organic fertilizer and in sandy soils applied with low rate of organic fertilizer, the adaptable varieties are variety *Soper* 9 *agritan* (V9); while in mega-environment 2, i.e. tidal swamplands and sandy soil applied with high rate organic fertilizer, variety *Bioguma agritan* (V11) are the most adaptive variety.

**Table 9.** Contrast comparison test on mean forage yield among varieties.

contrast	estimate	SE	df	lower.CL	Upper.CL	t.ratio	p.value
V3 vs V4 in E1	1.05	24	142	-46.39	48.5	0.044	0.9650
V3 vs V4 in E2	73.21	24	142	25.77	120.7	3.050	0.0027
V3 vs V4 in E6	43.47	24	142	-3.98	90.9	1.811	0.0722
V4 vs V9 in E1	31.06	24	142	-16.39	78.5	1.294	0.1978
V4 vs V9 in E2	-10.40	24	142	-57.85	37.0	-0.433	0.6653
V4 vs V9 in E6	10.88	24	142	-36.57	58.3	0.453	0.6510
V11 vs others in E3	35.83	39	142	-41.26	112.9	0.919	0.3597
V11 vs others in E4	-55.28	39	142	-132.38	21.8	-1.418	0.1585
V11 vs others in E5	92.36	39	142	15.27	169.5	2.368	0.0192

Figure 13 analyses the discriminativeness and representativeness of all tested environments.  Figure 14 gives the rank of the selected environment. Using the same reasoning as in the GGE biplot on grain yield, the tested environment E2 is the most discriminative and representative environment compared to the average environment. Therefore, tidal swampland applied with a high rate of organic fertilizer during the rainy season (E2) is ideal for selecting broadly adaptive genotypes/varieties based on forage yield (FY).

## 4. Discussion

MGIDI incorporates trait information into a single value to rank varieties or variety-environment combinations based on their distance from an “ideal type.” The “ideal type” or “ideotype” is a hypothetical variety/variety-environment combination with the best possible value for each trait. It has been successfully applied to several studies to enhance the performance, productivity, quality, or adaptability of different crops. ^14^ Each trait is assigned a weight based on its value or desirability, whereas superior varieties are those with the smallest distances from the ideal variety. The advantage of the MGIDI-based selection is that it incorporates several traits into the study and reduces the dimensions of these traits to just four factors that account for a significant portion of the variation. Finding varieties like ideotype types can be aided by the strengths and weaknesses of the selected varieties, as indicated by the contribution of each factor to the MGIDI. A helpful indicator for sorghum breeding or crop improvement would be the factors and their associated traits that contribute to the MGIDI of the selected varieties.

In contrast to the MGIDI, the GGE biplot tools only consider one trait at a time. In the GGE biplot technique applied in this study, we consider two valuable beneficial traits: grain yield and forage yield. The GGE biplot offers a more comprehensive evaluation of the best varieties across various environments (mega-environments). Furthermore, the ideal environment for identifying adaptable varieties, i.e., environments with representative and high-discriminating power, can be determined using the GGE biplot. Aside from the difference between MGIDI and GGE biplots, particularly in the traits they evaluate, comparing the results of the two methods in identifying the best varieties is worthwhile.

The GGE biplot has identified *Soper* 7 *Agritan* (V7) and *Numbu* (V8) as the top-performing varieties in terms of mean grain yield across various environments. These results differ somewhat from the varieties selected by the MGIDI, specifically V8 and V10. While V8 was chosen by the MGIDI and is acknowledged in the GGE biplot for its high mean grain yield, V10 was also selected by the MGIDI but does not perform as strongly in the GGE biplot, despite maintaining a mean grain yield above the average. Conversely, V7, not selected by the MGIDI, stands out as the top performer in grain yield according to the GGE biplot.

These discrepancies can be attributed to the fact that the contribution of FA1, which is associated with grain yield, is relatively small within the MGIDI for V8, thereby limiting its role in determining the highest mean grain yield. In contrast, the contribution of FA4, which does not relate to grain yield, is also minimal for V10 in the MGIDI. This difference implies that while V8 has significant value in terms of grain yield, V10 does not, although it may possess other traits related to FA4 that are unrelated to grain yield. The GGE biplot focuses solely on grain yield, which is why V7 was selected over V10.

The GGE biplot also shows the environment in which the varieties performed best in terms of their mean grain yield. During the rainy season, the *Soper* 7 agritan 7 (V7) variety is suitable for use in tidal swamplands during the wet season with either high-rate (E2) or low-rate organic fertilizers (E1), as well as in sandy soils with high-rate organic fertilizers (E6). Conversely, the *Numbu* (V8) variety is recommended for tidal swamplands during the dry season, particularly with high-rate (E4) or low-rate organic fertilizers (E3) and in sandy soils with low-rate organic fertilizers (E5). The MGIDI analysis indicated that variety V7 is also selected in the E2 environment and E3 environment, while V8 is selected in almost all environments except E1 and E3. These differences indicate that in specific environments (E1 and E2), V8 exhibits traits beyond grain yield that make it the closest to the ideal variety.

The highest means across environments have also been identified via the GGE biplot on forage yield (FY), using a logic like that of the GGE biplot on grain yield (GY). Like the grain yield, varieties differ from those chosen using the MGIDI. The Bioguma (V11) variety has the highest mean in tidal swamplands during both the rainy (E2) and dry seasons (E4), as well as in sandy soil (E6), when using high-rate organic fertilizer. Meanwhile, the *Soper* 9 agritan (V9) variety has the highest mean in tidal swamplands in both the wet and dry seasons, with a low rate of organic fertilizer (E1 and E3), as well as in sandy soil during the dry season, with a low rate of organic fertilizer (E1, E3, and E5). These differences indicated a variation in selecting grain yield and forage yield. Some varieties, however, are dual-purpose varieties, i.e., higher in grain yield as well as forage yield mean.

GGE biplot also determined the stability of the varieties in each group of environments. *Soper* 7 *agritan* (V7) and *Numbu* (V8) varieties that have the highest mean on grain yield, and *Bioguma* (V11) and *Soper* 9 *Agritan* (V9), which also have the highest mean in forage yield in their respective environments, are also relatively stable or have low variety-environment interactions. Therefore, they are adaptable varieties with the highest phenotypic mean (grain yield and forage yield) in the respective environments. Specifically, *Soper* 7 *Agritan* (V7) is adaptable in Mega-environment 1, and *Numbu* (V8) is adaptable in Mega-environment 2, as indicated by the GGE biplot on grain yield. At the same time, *Soper* 9 Agritan (V9) is adaptable in Mega-environment 1, and Bioguma (V11) is adaptable in Mega-environment 2, as shown in the GGE biplot for forage yield. The MGIDI, however, cannot identify adaptable varieties. The selection of a Variety-Environment combination can only determine which variety has the highest ranking in MGIDI. However, the selected variety-environment combination indicated that most varieties have a high MGIDI ranking, hence being close to ideal genotypes in environments E6, E4, and E2, which is like the result of the GGE biplot on forage yield.

The best environments for choosing broadly adaptive varieties could also be identified using the GGE biplot. These environments include tidal swamplands that are fertilized with a high rate of organic fertilizer during the rainy season (E2) to maximize forage yield and sandy soil that is fertilized with a high rate of organic fertilizer during the dry season (E6) to enhance grain yield. A high level of organic fertilizer enhances the environment’s ability to discriminate and represent the average environment. ^52, 53^ High rates of organic fertilizer have a significant impact on crops in tidal swamplands during the rainy season because they increase the populations of facultative and anaerobic microbes, which can help slow down the release of nutrients, add organic matter that can help bind particles in otherwise waterlogged areas, and help microbes release nutrients from organic material. In contrast, organic fertilizer increases fertility in sandy soils during the dry season by releasing nutrients slowly, a process that is particularly important in nutrient-poor sands. This process improves biological activity and soil life while also reducing compaction and erosion. These conditions will enhance environmental productivity, particularly for responsive varieties, thereby increasing the discriminating power of the environment.

A limitation of this study is that the tested environments are not sufficiently varied, so adaptability is not significantly broad. Planting seasons (dry and rainy season), agroecosystem type (tidal swamplands and sandy soils), and the rates of organic fertilizer application determine the environmental variations. Because the aim of this study is to find sorghum varieties for expansion of their cultivation to sandy soils and tidal swamplands, where organic fertilizer and planting seasons are crucial factors, such environmental variations are justified. Organic fertilizers significantly enhance the productivity and sustainability of agricultural practices in tidal swamplands and sandy soil. They are vital for improving soil fertility, ^20, 21^ enhancing crop productivity, ^54^ reducing environmental impacts, ^55^ and supporting sustainable agricultural practices in tidal swamps. ^56^ Combined with traditional knowledge and an integrated farming system, their use can transform these marginal lands into productive agricultural areas. The expansion of sorghum farming to tidal swamplands should consider using fertilizer and soil amelioration to improve soil fertility.

It might be possible to broaden the tested environment by extending the testing conditions to include different soil types, like peat soil, and different kinds of swamplands, like inland swamps, which might be able to support sorghum production, as well as different agronomic interventions. However, it might not be possible to expand testing conditions to waterlog and salinity pressures since sorghum is cultivated in type C and type D of tidal swamplands, which are unaffected by sea tide, and salinity stresses are only felt in type A tidal swamplands due to sea water intrusion. Expanding sorghum production in arid and upland regions may necessitate adaptability to heat stress and elevation changes. Adaptation of sorghum crops to heat stress ^7, 23, 24^ and different altitude ^27^ is possible, however testing such adaptability are beyond the scope of the current research.

## 5. Conclusion

Adaptable varieties differ for various groups of environments and different traits under consideration. Optimal environments for identifying broadly adaptable varieties varied by traits. The multitrait genotype-ideotype distance index proves to be a valuable tool for selecting varieties based on multiple traits. In parallel, the genotype plus genotype interaction biplot effectively identifies adaptable varieties based on individual trait.

## Ethics and consent

Ethical approval and consent were not required for this study, as it did not involve human participants, animal subjects, or sensitives data. The research focused on analyzing experimental data using publicly available software.

## Data Availability

The data underlying this study are available in Figshare at https://doi.org/10.6084/m9.figshare.29364263 for data excell (multitraits observation on sorghum) ^57^ and https://doi.org/10.6084/m9.figshare.29497829 for R code. ^58^ Data are available under the terms of the Creative Commons Attribution 4.0 International license (CC-BY 4.0).
