# Supplementary material for: Identifying Adaptable Varieties of Sorghum ( Sorghum bicolor L) in Tidal Swamplands and Sandy Soils by MGIDI and GGE Biplots
Source: F1000Res. 2026 Apr 15;14:883. Originally published 2025 Sep 9. [Version 3] doi: 10.12688/f1000research.166848.3 (PMC13142246; doi:10.12688/f1000research.166848.3)
Supplement: Peer review history

# Reviewer report: Reviewer response for version 3

**DOI:** 10.5256/f1000research.197679.r475376

**Published:** 2026-04-30

**Authors:** Niranjan Ravindra Thakur

**Affiliations:** 1 International Crops Research Institute for the Semi-Arid Tropics (ICRISAT), Hyderabad, Telangana, India

The paper is well-revised. No new comments.

Is the work clearly and accurately presented and does it cite the current literature?

Partly

If applicable, is the statistical analysis and its interpretation appropriate?

Partly

Are all the source data underlying the results available to ensure full reproducibility?

Yes

Is the study design appropriate and is the work technically sound?

Yes

Are the conclusions drawn adequately supported by the results?

Partly

Are sufficient details of methods and analysis provided to allow replication by others?

Partly

Reviewer Expertise:

Genetics and Plant Breeding

I confirm that I have read this submission and believe that I have an appropriate level of expertise to confirm that it is of an acceptable scientific standard.

# Reviewer report: Reviewer response for version 2

**DOI:** 10.5256/f1000research.189982.r443549

**Published:** 2026-02-26

**Authors:** Niranjan Ravindra Thakur

**Affiliations:** 1 International Crops Research Institute for the Semi-Arid Tropics (ICRISAT), Hyderabad, Telangana, India

1. Is the work clearly and accurately presented and does it cite the current literature?

**Partly**. The manuscript maintains a logical flow and identifies clear objectives; however, several typographical errors in the abstract and methodology sections detract from its precision. For example, the use of "multipl traits" and "were differed" in the abstract requires immediate correction to meet academic standards. Furthermore, while the literature review covers recent developments in MGIDI and GGE methodologies, it lacks a sufficiently deep integration of current research regarding the specific physiological challenges of tidal swamplands (e.g., aluminum toxicity, iron toxicity, and pyrite oxidation) which are critical for characterizing the "type C" and "type D" environments mentioned.

 2. Is the study design appropriate and is the work technically sound?

**Yes**. 

 3. Are sufficient details of methods and analysis provided to allow replication by others?

**Partly**. While the authors provide R code and specify the versions of software used, certain agronomic and soil details are missing. Specifically, a detailed physicochemical characterization of the soil at each experimental site prior to fertilization is absent, which is essential for defining the baseline stress levels. Additionally, there is an inconsistency in the description of plot dimensions (8 rows of 5m vs. 16 rows of 4m) which complicates the calculation of effective plot area and yield per hectare.

 4. If applicable, is the statistical analysis and its interpretation appropriate?

**Partly**. The use of MGIDI for multi-trait selection is an advanced and appropriate method that handles multicollinearity effectively. However, the GGE biplot interpretation relies on an environment-focused singular value partitioning (SVP=2) for genotype evaluation. In established GGE biplot theory, a genotype-focused partitioning (SVP=1) is generally required to accurately visualize the relationships and distances among genotypes. The modification of the R code in version 2 to include contrast comparisons (Tables 8 and 9) helps bridge this gap but does not fully resolve the graphical inconsistency.

 5. Are all the source data underlying the results available to ensure full reproducibility?

**Yes**.

 6. Are the conclusions drawn adequately supported by the results?

**Partly**. The selection of Soper 7 and Numbu as adaptable for grain yield is well-supported by the empirical data in Table 7 and the biplots. However, the conclusion that these results can guide "broadly" adaptable varieties for all tidal swamplands and sandy soils is overextended. The results are province-specific and do not account for the high variability in salinity or waterlogging intensity that exists across the 8.92 million hectares of tidal swamplands in Indonesia.

 Other comments:

1. A meticulous audit of the mathematical expressions in the manuscript reveals several points of concern that require rectification to ensure scientific rigor. In Equation 1, there is a fundamental notation error: the term E _l_ represents the environmental effect, but the index l is not linked to the i environment mentioned in the subscript of Y _ijkt_. To maintain consistency, this should be written as E _i_.
2. Kindly verify the MGIDI formula according to the standard MGIDI literature (Olivoto and Nardino, 2020) https://doi.org/10.1093/bioinformatics/btaa981.
3. Section 2.4.4.: The manuscript specifies that biplots were constructed at f=0 (SVP=2). The SVP=2 is appropriate for environmental evaluation (column metric preserving), it is not the ideal setting for visualizing distances between genotypes (row metric preserving), which requires f=1 (SVP=1). The authors should justify why environment-focused partitioning was used for genotype ranking or provide the genotype-focused plots as supplementary material.
4. It would be nice to add the relevance of the traits studied. For instance, trait code “BRIX” has relevance for “Measures soluble solids (sugar) in the stem for bioenergy potential.”, similarly, “PH” has, “Essential for biomass estimation and lodging risk assessment.”.
5. The current manuscript is noted as having a "partly" adequate discussion that lacks deep integration of the specific physiological challenges of tidal swamplands, such as acidity or nutrient-poor conditions. These insights provide the authors with a template to explain why certain varieties succeeded, rather than just stating that they succeeded.
6. The observation that high-fertilizer environments (1000 kg/ha) were significantly more discriminating than their 500 kg/ha counterparts, as it improved the discriminating power of the trials, is a major finding. This suggests that in nutrient-impoverished soils like Inceptisols and Entisols, severe resource limitation can mask genotypic variance, leading to "false stability" where varieties appear similar simply because they are all surviving at a metabolic minimum. Authors should be prompted to discuss how resource limitation in marginal soils can mask genotypic variance, a critical concept for breeding in stress-prone environments.
7. The functional divergence between grain yield specialists (like Soper 7) and forage yield specialists (like Bioguma II) suggests a significant physiological trade-off in biomass allocation. Forcing the authors to discuss these trade-offs makes their variety recommendations more scientifically robust and practically useful for different agricultural sectors (food vs. silage).
8. Explicitly discussing why Numbu was selected across multiple methodologies (MGIDI and GGE) while others were not helps justify the use of advanced indices like MGIDI for selecting "resilient" rather than just "high-yielding" genotypes.
9. While the authors used modern tools, the integration of additional methodologies could provide even deeper insights into the adaptability of these varieties. This includes (A) Linear Mixed Model-based approaches: Recent literature suggests that Linear Mixed Model-based approaches, particularly Factor Analytic (FA) models, are superior for capturing complex spatial variation and handling heterogeneous variances across environments. (B) SpATS: Integrating a two-dimensional P-spline mixed model (SpATS) would allow the researchers to account for localized field trends within the tidal swamplands and sandy soils. Given the likely variability in water table levels in type C swamps, spatial analysis could differentiate between true genetic performance and environmental noise caused by drainage micro-gradients. (C) Risk and Probability: By calculating the joint probability of superior performance and yield stability, the researchers could offer farmers a quantitative measure of risk for each variety (e.g., "Variety H2 has a 99% probability of belonging to the top subset in sandy soils"). This approach provides actionable decision-making data beyond traditional stability indices. (D) Genomic Selection and Sparse Testing: For future breeding cycles, the authors might consider sparse testing designs combined with genomic prediction. This methodology optimizes resource allocation by evaluating overlapping genotypes across environments, allowing for the inference of unobserved genotype-in-environment combinations, thereby reducing phenotyping costs in remote regions like Central Kalimantan.
10. Abstract Correction: The term "multipl traits" must be corrected to "multiple traits" and "were differed" should be revised to "differed" or "were different" to improve readability.
11. Plot Dimension Consistency: Authors must clarify whether each plot consisted of 8 rows (5m long) or 16 rows (4m long). This is vital for calculating yield per unit area.
12. Initial Soil Data: Provide a summary table of the initial soil physicochemical properties (pH, EC, C-organic, N-total, P-available, K-exchangeable, Al-saturation, and other micronutrients like Fe and Zn) for both locations to characterize the baseline stress levels.
13. Formula Verification: Check and correct all the equations given in the manuscript.
14. SVP Logic in GGE: Justify the use of environment-focused partitioning (SVP=2) for genotype evaluation, as genotype-focused partitioning (SVP=1) is standard for visualizing inter-genotypic relationships.
15. Weather Summary: Include a summary of rainfall and temperature data for the trial sites during the 2022-2024 period to support the planting season (wet vs. dry) classifications.
16. Variety History: Briefly describe the breeding history (pedigree) or known parents for tested varieties to reduce the "na" (not available) data points in Table 1.
17. Trait Selection Weights: Clarify the rationale for the specific weights assigned to traits in MGIDI (e.g., PH: 0.4, GY: 1.0). Were these weights based on economic value or expert breeder judgment?
18. MGIDI and GGE Comparison: Explicitly discuss why Soper 7 (V7) was selected by GGE for yield but not by MGIDI, and how this affects the final variety recommendation.

Is the work clearly and accurately presented and does it cite the current literature?

Partly

If applicable, is the statistical analysis and its interpretation appropriate?

Partly

Are all the source data underlying the results available to ensure full reproducibility?

Yes

Is the study design appropriate and is the work technically sound?

Yes

Are the conclusions drawn adequately supported by the results?

Partly

Are sufficient details of methods and analysis provided to allow replication by others?

Partly

Reviewer Expertise:

Genetics and Plant Breeding

I confirm that I have read this submission and believe that I have an appropriate level of expertise to confirm that it is of an acceptable scientific standard, however I have significant reservations, as outlined above.

# Response

**Published:** 2026-03-12

**Authors:** Muhamad Sabran Sabran

**Affiliations:** Research Center for Food Crops, National Research and Innovation Agency, Cibinong, Jawa Barat, Indonesia

1. Is the work clearly and accurately presented and does it cite the current literature?
  Partly. The manuscript maintains a logical flow and identifies clear objectives; however, several typographical errors in the abstract and methodology sections detract from its precision. For example, the use of "multipl traits" and "were differed" in the abstract requires immediate correction to meet academic standards. Furthermore, while the literature review covers recent developments in MGIDI and GGE methodologies, it lacks a sufficiently deep integration of current research regarding the specific physiological challenges of tidal swamplands (e.g., aluminum toxicity, iron toxicity, and pyrite oxidation) which are critical for characterizing the "type C" and "type D" environments mentioned.

Response: abstract has been revised and Physiological challenges in tidal swamplands are added in introduction section.

 2. Is the study design appropriate and is the work technically sound?

 Yes. 

 3. Are sufficient details of methods and analysis provided to allow replication by others?

 Partly. While the authors provide R code and specify the versions of software used, certain agronomic and soil details are missing. Specifically, a detailed physicochemical characterization of the soil at each experimental site prior to fertilization is absent, which is essential for defining the baseline stress levels. Additionally, there is an inconsistency in the description of plot dimensions (8 rows of 5m vs. 16 rows of 4m) which complicates the calculation of effective plot area and yield per hectare.

 Response ; detailed physicochemical characterization of the soil at each experimental site prior to fertilization has been added and the plot size clarified in material and method section.

 4. If applicable, is the statistical analysis and its interpretation appropriate?

 Partly. The use of MGIDI for multi-trait selection is an advanced and appropriate method that handles multicollinearity effectively. However, the GGE biplot interpretation relies on an environment-focused singular value partitioning (SVP=2) for genotype evaluation. In established GGE biplot theory, a genotype-focused partitioning (SVP=1) is generally required to accurately visualize the relationships and distances among genotypes. The modification of the R code in version 2 to include contrast comparisons (Tables 8 and 9) helps bridge this gap but does not fully resolve the graphical inconsistency.

 Response : 

 the GGE biplot has been revised, the genotype_based parttioning was used for the “which-won-were”,”mean and stability”, and “variety Ranking” biplots (figure 2a-c, and figure 3a-c), while environment base partitioning(svp=2)was used for discriminitavenss, environment relation, and environment ranking. (figure 2d-f and figure 3d-f

 5. Are all the source data underlying the results available to ensure full reproducibility?

 Yes.

 6. Are the conclusions drawn adequately supported by the results?

 Partly. The selection of Soper 7 and Numbu as adaptable for grain yield is well-supported by the empirical data in Table 7 and the biplots. However, the conclusion that these results can guide "broadly" adaptable varieties for all tidal swamplands and sandy soils is overextended. The results are province-specific and do not account for the high variability in salinity or waterlogging intensity that exists across the 8.92 million hectares of tidal swamplands in Indonesia.

 Response:

 Agreed. We revised the coclusion accordingly

 Other comments:

1. A meticulous audit of the mathematical expressions in the manuscript reveals several points of concern that require rectification to ensure scientific rigor. In Equation 1, there is a fundamental notation error: the term El represents the environmental effect, but the index l is not linked to the i environment mentioned in the subscript of Yijkt. To maintain consistency, this should be written as Ei.

Response : the mathematical expression has been revised accordingly

1. Kindly verify the MGIDI formula according to the standard MGIDI literature (Olivoto and Nardino, 2020) https://doi.org/10.1093/bioinformatics/btaa981.

Response  the MGIDI formula has been verified according standard MGIDI literature. citation added

1. Section 2.4.4.: The manuscript specifies that biplots were constructed at f=0 (SVP=2). The SVP=2 is appropriate for environmental evaluation (column metric preserving), it is not the ideal setting for visualizing distances between genotypes (row metric preserving), which requires f=1 (SVP=1). The authors should justify why environment-focused partitioning was used for genotype ranking or provide the genotype-focused plots as supplementary material.

Response: Section 2.4.4 has been revised

1. It would be nice to add the relevance of the traits studied. For instance, trait code “BRIX” has relevance for “Measures soluble solids (sugar) in the stem for bioenergy potential.”, similarly, “PH” has, “Essential for biomass estimation and lodging risk assessment.”.

Response : The relevance traits added in column 3 tabel 1

1. The current manuscript is noted as having a "partly" adequate discussion that lacks deep integration of the specific physiological challenges of tidal swamplands, such as acidity or nutrient-poor conditions. These insights provide the authors with a template to explain why certain varieties succeeded, rather than just stating that they succeeded.

Response : integration with specific challenges in tidal swamplands added in the discussion

1. The observation that high-fertilizer environments (1000 kg/ha) were significantly more discriminating than their 500 kg/ha counterparts, as it improved the discriminating power of the trials, is a major finding. This suggests that in nutrient-impoverished soils like Inceptisols and Entisols, severe resource limitation can mask genotypic variance, leading to "false stability" where varieties appear similar simply because they are all surviving at a metabolic minimum. Authors should be prompted to discuss how resource limitation in marginal soils can mask genotypic variance, a critical concept for breeding in stress-prone environments.

Resoponse

 This was discussed briefly in paragraph 8 im the “Discussion Section”

1. The functional divergence between grain yield specialists (like Soper 7) and forage yield specialists (like Bioguma II) suggests a significant physiological trade-off in biomass allocation. Forcing the authors to discuss these trade-offs makes their variety recommendations more scientifically robust and practically useful for different agricultural sectors (food vs. silage).

 

1. Explicitly discussing why Numbu was selected across multiple methodologies (MGIDI and GGE) while others were not helps justify the use of advanced indices like MGIDI for selecting "resilient" rather than just "high-yielding" genotypes.

Response: paragraph 10 in the discussion has been added “Stronger vegetative growth and improved tolerance to acidic soil conditions and nutrition limitations, such as in tidal swamplands and sandy soils, contributed to Numbu's comparatively consistent performance…..etc” to explain why Numbu selected across multiple methodologies while others is not.

1. While the authors used modern tools, the integration of additional methodologies could provide even deeper insights into the adaptability of these varieties. This includes (A) Linear Mixed Model-based approaches: Recent literature suggests that Linear Mixed Model-based approaches, particularly Factor Analytic (FA) models, are superior for capturing complex spatial variation and handling heterogeneous variances across environments. (B) SpATS: Integrating a two-dimensional P-spline mixed model (SpATS) would allow the researchers to account for localized field trends within the tidal swamplands and sandy soils. Given the likely variability in water table levels in type C swamps, spatial analysis could differentiate between true genetic performance and environmental noise caused by drainage micro-gradients. (C) Risk and Probability: By calculating the joint probability of superior performance and yield stability, the researchers could offer farmers a quantitative measure of risk for each variety (e.g., "Variety H2 has a 99% probability of belonging to the top subset in sandy soils"). This approach provides actionable decision-making data beyond traditional stability indices. (D) Genomic Selection and Sparse Testing: For future breeding cycles, the authors might consider sparse testing designs combined with genomic prediction. This methodology optimizes resource allocation by evaluating overlapping genotypes across environments, allowing for the inference of unobserved genotype-in-environment combinations, thereby reducing phenotyping costs in remote regions like Central Kalimantan.

Response 

 Agreed. This was briefly added in the discussion for future research.

1. Abstract Correction: The term "multipl traits" must be corrected to "multiple traits" and "were differed" should be revised to "differed" or "were different" to improve readability.

Response : abstract revised

1. Plot Dimension Consistency: Authors must clarify whether each plot consisted of 8 rows (5m long) or 16 rows (4m long). This is vital for calculating yield per unit area.

Plot size : revised and clarified

1. Initial Soil Data: Provide a summary table of the initial soil physicochemical properties (pH, EC, C-organic, N-total, P-available, K-exchangeable, Al-saturation, and other micronutrients like Fe and Zn) for both locations to characterize the baseline stress levels.

Initial daya provided

1. Formula Verification: Check and correct all the equations given in the manuscript.

Formula verified

1. SVP Logic in GGE: Justify the use of environment-focused partitioning (SVP=2) for genotype evaluation, as genotype-focused partitioning (SVP=1) is standard for visualizing inter-genotypic relationships.

The biplot has been revised, svp=1 was used to study the difference in genotype while svp=2 for study the environment

1. Weather Summary: Include a summary of rainfall and temperature data for the trial sites during the 2022-2024 period to support the planting season (wet vs. dry) classifications.

Rainfall has been summarized 

1. Variety History: Briefly describe the breeding history (pedigree) or known parents for tested varieties to reduce the "na" (not available) data points in Table 1.

Variety History provided

1. Trait Selection Weights: Clarify the rationale for the specific weights assigned to traits in MGIDI (e.g., PH: 0.4, GY: 1.0). Were these weights based on economic value or expert breeder judgment?

Weight assignment clarified

1. MGIDI and GGE Comparison: Explicitly discuss why Soper 7 (V7) was selected by GGE for yield but not by MGIDI, and how this  affects the final variety recommendation.

Respon MGIDI considered many traits simultaneously not only grain yield or forage yield in GGE biplot

# Reviewer report: Reviewer response for version 2

**DOI:** 10.5256/f1000research.189982.r441180

**Published:** 2026-02-13

**Authors:** Tushar Arun Mohanty

**Affiliations:** 1 Tamil Nadu Agricultural University, Coimbatore, Tamil Nadu, India

**1. Abstract :** **Comment 1: Clarity and Structure**

 The abstract clearly mentions the objectives, methodologies (MGIDI and GGE), and major findings. However, the sentence construction in some portions is grammatically inconsistent and needs polishing for better readability.

**Comment 2: Quantitative Support**

 The abstract reports selected varieties but does not provide numerical performance indicators (e.g., % superiority, MGIDI values, yield differences). Including key quantitative results would strengthen scientific precision.

**Comment 3: Conclusion Alignment**

 The conclusion in the abstract broadly summarizes adaptability but could better align with the specific environments (E1–E6) tested

**2. Introduction**

**Comment 1: Relevance and Literature**

 The introduction is supported by relevant and recent references on sorghum utilization and adaptability. The rationale for using MGIDI and GGE biplot is appropriate

**Comment 2: Justification of Study**

 The justification for targeting tidal swamplands (type C & D) and sandy soils is logical but could be supported with more quantitative background on production constraints.

**Comment 3: Research Gap**

 The manuscript could more explicitly state the research gap—especially regarding multi-trait selection in marginal Indonesian agroecosystems.

**3. Materials and Methods**

**3.1 Experimental Sites and Design**

**Comment 1: Environmental Description**

 The environments (E1–E6) are clearly defined with fertilizer levels and seasons. However, detailed soil physicochemical properties are not adequately presented.

**Comment 2: Experimental Design**

 RCBD with three replications is appropriate. Plot size description appears inconsistent (8 × 5 m rows and 16 × 4 m rows) and requires clarification.

**Comment 3: Agronomic Management**

 Basic crop management practices are described, but more detail on irrigation regime and pest management would improve reproducibility.

**3.2 Plant Material**

**Comment 1: Variety Description**

 The 12 varieties are well documented with origin and resistance traits. However, missing data (e.g., “na” for tannin/yield) should be explained statistically.

**Comment 2: Genetic Background**

 More discussion on genetic diversity among varieties would strengthen the biological relevance of MGIDI application.

**3.3 Data Analysis**

**Comment 1: Statistical Model**

 The MANOVA model is clearly presented. However, assumptions (normality, homogeneity) are not discussed.

**Comment 2: MGIDI Methodology**

 Rescaling and matrix construction are mentioned, but factor retention criteria and missing data treatment should be described more explicitly.

**Comment 3: GGE Biplot**

 Use of PC1 and PC2 from environment-centered data is appropriate. Still, justification of variance explained by PCs should be clearly reported.

**Comment 4: Statistical Rigor**

 The manuscript relies heavily on graphical interpretation. Numerical validation (confidence intervals, standard errors, contrast tests) should be emphasized.

**4. Results**

**Comment 1: MGIDI Selection**

 Selection of Numbu and Kawali based on MGIDI average ranking is clearly stated. However, actual MGIDI values and selection intensity are not highlighted sufficiently.

**Comment 2: G×E Interaction**

 Significant G×E interaction is acknowledged, but variance components or % contribution are not clearly quantified.

**Comment 3: Trait-Specific Adaptability**

 Soper 7 and Numbu show adaptability for grain weight and forage yield. Trait-specific ranking tables would improve clarity.

**Comment 4: Environmental Interpretation**

 Identification of high organic fertilizer environments and sandy soils as optimal selection sites is meaningful but needs agronomic explanation.

**5. Discussion**

**Comment 1: Interpretation Depth**

 The discussion links findings to adaptability concepts but could further integrate global sorghum adaptability research.

**Comment 2: Environmental Scope**

 The study acknowledges limited environmental heterogeneity (two soil types, two fertilizer levels). This remains a limitation for broader generalization.

**Comment 3: Statistical Limitations**

 Potential Type I error, multicollinearity among traits, and dimensionality reduction effects should be discussed more critically.

**6. Conclusion**

**Comment 1: Logical Consistency**

 The conclusion aligns with results. It appropriately emphasizes MGIDI and GGE as complementary tools.

**Comment 2: Strength of Claims**

 Statements regarding “valuable tool” should be slightly moderated considering limited environmental testing.

**Comment 4: Future Research**

 Recommendations for broader G×Management studies are appropriate but could be elaborated with specific design suggestions.

 This manuscript evaluates 12 sorghum varieties across six environments (tidal swamplands and sandy soils) using MGIDI and GGE biplot to identify adaptable genotypes. The design is sound and data transparent. However, limited environmental diversity and insufficient statistical detailing reduce generalizability. Minor methodological clarification and stronger quantitative reporting are recommended before indexing.

Is the work clearly and accurately presented and does it cite the current literature?

Yes

If applicable, is the statistical analysis and its interpretation appropriate?

Partly

Are all the source data underlying the results available to ensure full reproducibility?

Yes

Is the study design appropriate and is the work technically sound?

Yes

Are the conclusions drawn adequately supported by the results?

Partly

Are sufficient details of methods and analysis provided to allow replication by others?

Yes

Reviewer Expertise:

Plant Breeder, Hybrid Rice, Twoline Hybrid Rice, Molecular genetics, Genetics, Sesame

I confirm that I have read this submission and believe that I have an appropriate level of expertise to confirm that it is of an acceptable scientific standard, however I have significant reservations, as outlined above.

# Response

**Published:** 2026-03-12

**Authors:** Muhamad Sabran Sabran

**Affiliations:** Research Center for Food Crops, National Research and Innovation Agency, Cibinong, Jawa Barat, Indonesia

**Abstract :**

 Comment 1: Clarity and Structure

 The abstract clearly mentions the objectives, methodologies (MGIDI and GGE), and major findings. However, the sentence construction in some portions is grammatically inconsistent and needs polishing for better readability.

 Response: some sentence construction has been revised

 Comment 2: Quantitative Support

 The abstract reports selected varieties but does not provide numerical performance indicators (e.g., % superiority, MGIDI values, yield differences). Including key quantitative results would strengthen scientific precision.

 Response: due to limited space in the abstracts (limited number of words) we prefer not to put numerical indicator on the selected varieties in the abstracts. However such indicators could be find everywhere in the results section

 Comment 3: Conclusion Alignment

 The conclusion in the abstract broadly summarizes adaptability but could better align with the specific environments (E1–E6) tested

       Response: the alignment with specific environment is given in the conclusion, however due to limited space in abstract, we summarized the the alignment of the stability with environment in the following sentence: “Adaptable varieties differ for various groups of environments and different traits under consideration”

 2. Introduction

 Comment 1: Relevance and Literature

 The introduction is supported by relevant and recent references on sorghum utilization and adaptability. The rationale for using MGIDI and GGE biplot is appropriate

 Response: no comment

 Comment 2: Justification of Study

 The justification for targeting tidal swamplands (type C & D) and sandy soils is logical but could be supported with more quantitative background on production constraints.

 Response: 

 Quantitative background on production constraint in tidal swampland and sandy soil has been added 

 Comment 3: Research Gap

 The manuscript could more explicitly state the research gap—especially regarding multi-trait selection in marginal Indonesian agroecosystems.

 Response :  

 Research gap has been added in the manuscript at paragraph 7 in the INTRODUCTION section

 3. Materials and Methods

 3.1 Experimental Sites and Design

 Comment 1: Environmental Description

 The environments (E1–E6) are clearly defined with fertilizer levels and seasons. However, detailed soil physicochemical properties are not adequately presented.

 Response: 

 Detailed soil physicochemical properties are presented in Experimental Sites and Design subsection in the Material and method section

 Comment 2: Experimental Design

 RCBD with three replications is appropriate. Plot size description appears inconsistent (8 × 5 m rows and 16 × 4 m rows) and requires clarification.

 Response:

 The plot size was 5x4 cm with planting distant between row 0.6 m and within row 0.25 m, i.e .there are 8 planting hole in 5m column and 16 planting hole in 4m row.

 Comment 3: Agronomic Management

 Basic crop management practices are described, but more detail on irrigation regime and pest management would improve reproducibility.

 Response: 

 “Irrigation by watering the plants with a hose for 4-5 hours during the early growth and seed filling. Fungicide with the active ingredients difenokonazol and azoksistrobin were used to control diseases caused by fungi, whereas insecticide with the active ingredient karbofuran was used to control pests” these sentences has been added in experimental design and observation section. 

 3.2 Plant Material

 Comment 1: Variety Description

 The 12 varieties are well documented with origin and resistance traits. However, missing data (e.g., “na” for tannin/yield) should be explained statistically.

 Response: the missing data has been found. Table has been revised

 Comment 2: Genetic Background

 More discussion on genetic diversity among varieties would strengthen the biological relevance of MGIDI application.

 Response:    the genetic diversity and genetic background has been added below table 1.

 3.3 Data Analysis

 Comment 1: Statistical Model

 The MANOVA model is clearly presented. However, assumptions (normality, homogeneity) are not discussed.

 Response:

 Yes the error (eij) is assumed to be multivariate normal with mean 0 and positive definite covariance matrix

 Comment 2: MGIDI Methodology

 Rescaling and matrix construction are mentioned, but factor retention criteria and missing data treatment should be described more explicitly.

 Response 

 Factors associated with eigenvalue of the correlation matrix greater than 1 are retained

 Comment 3: GGE Biplot

 Use of PC1 and PC2 from environment-centered data is appropriate. Still, justification of variance explained by PCs should be clearly reported.

 Responses

 The contribution PC1 + PC2 in this article is 86.60 % for grain yield and 94% for forage yield. Although there are no hard cutoff but generally contribution > 80%  is excellent. The results is clearly reported

 Comment 4: Statistical Rigor

 The manuscript relies heavily on graphical interpretation. Numerical validation (confidence intervals, standard errors, contrast tests) should be emphasized.

 Response:

 Contrast test and its confidence interval is given table 9

 4. Results

 Comment 1: MGIDI Selection

 Selection of Numbu and Kawali based on MGIDI average ranking is clearly stated. However, actual MGIDI values and selection intensity are not highlighted sufficiently.

 Response: 

 Selection intensity is 20%, the MDIGI values is given in table 7

 Comment 2: G×E Interaction

 Significant G×E interaction is acknowledged, but variance components or % contribution are not clearly quantified.

 Response: Variance components or % contribution is given in Table 10.

 Comment 3: Trait-Specific Adaptability

 Soper 7 and Numbu show adaptability for grain weight and forage yield. Trait-specific ranking tables would improve clarity.

 Response: Values of mgidi are given in table 7, while  grain yield and forage yield means are given  is given in table 9

 Comment 4: Environmental Interpretation

 Identification of high organic fertilizer environments and sandy soils as optimal selection sites is meaningful but needs agronomic explanation.

 Response.  

 These environments were selected to exert specific environmental selection pressures. The high-organic (swamp) soils screen for anaerobic tolerance and organic acid resilience, while sandy soils evaluate nutrient-use efficiency and root condition under drought-prone and high-leaching conditions. This ensures the identified genotypes possess the robust mechanisms necessary for productivity in marginal ecosystems.

 5. Discussion

 Comment 1: Interpretation Depth

 The discussion links findings to adaptability concepts but could further integrate global sorghum adaptability research.

 Response

 Research on sorghum adaptation worldwide mostly focuses on low fertility soils and resilience to heat and drought. Two underrepresented agro-ecosystems—sandy soils and tidal swamplands—will be added by this project. Additionally, it can be incorporated into global frameworks for adaption breeding that involve multi-environmental trials. Breeding pipelines for stress tolerance can be strengthened by the contribution of adaptive alleles for wet tolerance and sandy soil resilience from genotypes that are stable across severe soils, such as Numbu, Kawali, and Soper 7 Agritan.

 Comment 2: Environmental Scope

   The study acknowledges limited environmental heterogeneity (two soil types, two fertilizer levels). This remains a limitation for broader generalization.

 Comment 3: Statistical Limitations

 Potential Type I error, multicollinearity among traits, and dimensionality reduction effects should be discussed more critically.

 Multicollinearity among agronomic and physiological traits can bias multi-trait selection indices by inflating the weight of correlated trait groups. To overcome this limitation, the MGIDI approach applies factor analysis to reduce dimensionality and transform correlated variables into orthogonal latent factors. This ensures balanced representation of independent biological processes and prevents dominance of highly correlated productivity traits, thereby enhancing the reliability of genotype ranking under multi-stress environments."

 6. Conclusion

 Comment 1: Logical Consistency

 The conclusion aligns with results. It appropriately emphasizes MGIDI and GGE as complementary tools.

 “MGIDI and GGE biplot provide complementary perspectives for genotype evaluation. While MGIDI identifies genotypes closest to the ideotype by integrating multiple correlated traits into orthogonal factors, GGE biplot elucidates genotype performance patterns across environments, revealing stability, mega-environment structure, and specific adaptation. Their combined application enables robust selection of superior genotypes with both multi-trait superiority and wide environmental adaptability.”

 Comment 2: Strength of Claims

 Statements regarding “valuable tool” should be slightly moderated considering limited environmental testing.

 Response

 The claim have been moderated by the following statement:”When choosing varieties based on numerous taits, the MGIDI  might be a useful tool. However, environmental testing's limitations could make it less effective”.

 Comment 4: Future Research

 Recommendations for broader G×Management studies are appropriate but could be elaborated with specific design suggestions.

 Responses

 No explicit statement on broader GXManagement studies. However it was implicitly stated at the end of discussion session.

# Reviewer report: Reviewer response for version 2

**DOI:** 10.5256/f1000research.189982.r430443

**Published:** 2025-11-24

**Authors:** Ramendra Nath Sarma

**Affiliations:** 1 Assam Agricultural University, Assam, India

Thank you for the opportunity to review the revised version of the manuscript by Susilawati et al. I have examined the authors' responses and the changes made to the manuscript.

 I am pleased to note that the authors have made commendable efforts to address the points raised in the initial review. The revisions, particularly the inclusion of summary statistics in Table 7, the contrast comparisons in Tables 8 and 9, and the improved clarification of the statistical methodology in sections 2.4.1 and 2.4.4, have significantly strengthened the manuscript's clarity and analytical rigor. The expanded discussion now more appropriately acknowledges the scope of the study.

 While my primary concern regarding the limited number of test environments remains a notable limitation for the broad generalizability of the findings, the authors have adequately justified their experimental scope within the context of their research objectives. They have also appropriately framed this as a limitation and suggested valuable directions for future research to build upon these initial results.

 Considering the substantial improvements made in this revision and the manuscript's value in applying advanced statistical tools to sorghum cultivation in underutilized agroecologies, I recommend that the manuscript be accepted for Indexing in its current form.

Is the work clearly and accurately presented and does it cite the current literature?

Partly

If applicable, is the statistical analysis and its interpretation appropriate?

Partly

Are all the source data underlying the results available to ensure full reproducibility?

Yes

Is the study design appropriate and is the work technically sound?

Partly

Are the conclusions drawn adequately supported by the results?

No

Are sufficient details of methods and analysis provided to allow replication by others?

Partly

Reviewer Expertise:

Plant breeder

I confirm that I have read this submission and believe that I have an appropriate level of expertise to confirm that it is of an acceptable scientific standard.

# Reviewer report: Reviewer response for version 1

**DOI:** 10.5256/f1000research.183896.r413325

**Published:** 2025-09-18

**Authors:** Ramendra Nath Sarma

**Affiliations:** 1 Assam Agricultural University, Assam, India

The manuscript provides a thorough evaluation of sorghum varieties for adaptability in tidal swamplands and sandy soils using MGIDI and GGE biplots. It is well-structured with clear objectives, robust designs, and relevant contemporary citations, but there are areas for improvement regarding statistical presentation, environmental scope, and method transparency.

 1. Occasional typographical errors, formatting inconsistencies, and missing punctuation may hinder readability (e.g., sporadic placement of special characters, missing trait abbreviations).

 2. Environmental scope is narrow: Although justified as a limitation, two soil types and fertilizer treatments may not generalize to all marginal environments; additional environmental heterogeneity would strengthen the inference.

 3. Some method descriptions (e.g., specifics of matrix rescaling, factor retention criteria, missing data treatment, custom R scripts used) are too brief for reproduction by readers unfamiliar with MGIDI implementation in R.

 4. Lack of some summary statistics: Key results rely on graphical (biplot/polygon) interpretation, with few numerical summaries or confidence intervals for key rankings. Discussion of statistical limitations (e.g., potential for Type I error, treatment of marginally significant factors, or post hoc comparisons) is limited.

 5. Suggest that future research should test broader sets of environments and explicitly include analyses of genotype × management interactions—such as how sorghum varieties respond to combined agronomic interventions or more complex environmental stresses—to improve generalizability and guide breeding decisions for marginal lands.

 6. Discuss how factors such as greater variability in soil types, additional climatic stresses (e.g., salinity, waterlogging, temperature extremes), or alternative management practices (different fertilizer blends, irrigation regimes, tillage practices, intercropping systems) might influence genotype performance and adaptability beyond what was observed in the present work.

 In summary, the manuscript is a well-constructed, methodologically modern work that integrates recent literature and advanced statistical genetics tools in crop adaptability research. Weaknesses are mainly in the breadth of environments, clarity of some analytical details, and depth of statistical reporting, yet it achieves most criteria for academic merit, reproducibility, and result support expected in genetic crop adaptation studies.

Is the work clearly and accurately presented and does it cite the current literature?

Partly

If applicable, is the statistical analysis and its interpretation appropriate?

Partly

Are all the source data underlying the results available to ensure full reproducibility?

Yes

Is the study design appropriate and is the work technically sound?

Partly

Are the conclusions drawn adequately supported by the results?

No

Are sufficient details of methods and analysis provided to allow replication by others?

Partly

Reviewer Expertise:

Plant breeder

I confirm that I have read this submission and believe that I have an appropriate level of expertise to confirm that it is of an acceptable scientific standard, however I have significant reservations, as outlined above.

# Response

**Published:** 2025-10-19

**Authors:** Muhamad Sabran Sabran

**Affiliations:** Research Center for Food Crops, National Research and Innovation Agency, Cibinong, Jawa Barat, Indonesia

1. Occasional typographical errors, formatting inconsistencies, and missing punctuation may hinder readability (e.g., sporadic placement of special characters, missing trait abbreviations).

*Typographical errors, formatting inconsistencies, and missing punctuation has been improved in the new version of manuscript.*

 2. Environmental scope is narrow: Although justified as a limitation, two soil types version of the and fertilizer treatments may not generalize to all marginal environments; additional environmental heterogeneity would strengthen the inference.

*We agreed with the narrow scope of environmental heterogeneity, however since the objective is to identify varieties of sorghum for expansion of their cultivation tidal swamplands in which organic fertilizer and planting season are the determining factor, the limitation of the environmental variability is justified. In addition, expansion of sorghum cultivation in tidal swamplands is not possible to waterlogged condition such type A and B (direct influence of sea tide. Therefore, possible expansion is only to type C and D of tidal swamplands. To have a wider testing environment need to add agronomic interventions in Tidal swampland and sandy soil, which require new experiments.*

 3. Some method descriptions (e.g., specifics of matrix rescaling, factor retention criteria, missing data treatment, custom R scripts used) are too brief for reproduction by readers unfamiliar with MGIDI implementation in R.

*Matrix scaling, scoring and R code has been revised in the new version.*

 4. Lack of some summary statistics: Key results rely on graphical (biplot/polygon) interpretation, with few numerical summaries or confidence intervals for key rankings. Discussion of statistical limitations (e.g., potential for Type I error, treatment of marginally significant factors, or post hoc comparisons) is limited.

*Summary statistics in GGE biplots including contrast comparison test among variety in each environment has been added in the new version of the manuscript (table 7, table 8 and table 9).it will support the graphical biplot presentation.*

 5. Suggest that future research should test broader sets of environments and explicitly include analyses of genotype × management interactions—such as how sorghum varieties respond to combined agronomic interventions or more complex environmental stresses—to improve generalizability and guide breeding decisions for marginal lands.

*Suggestion for future research has been added in the revised version of the paper*

*(the last paragraph of the discussion section)*

 6. Discuss how factors such as greater variability in soil types, additional climatic stresses (e.g., salinity, waterlogging, temperature extremes), or alternative management practices (different fertilizer blends, irrigation regimes, tillage practices, intercropping systems) might influence genotype performance and adaptability beyond what was observed in the present work.

*This has been implicitly explain in  the last paragraph of the “Discussion section”*
